# Landscape of targeted therapies for advanced urothelial carcinoma

**DOI:** 10.37349/etat.2024.00240

**Published:** 2024-06-21

**Authors:** Shihao Shang, Lei Zhang, Kepu Liu, Maoxin Lv, Jie Zhang, Dongen Ju, Di Wei, Zelong Sun, Pinxiao Wang, Jianlin Yuan, Zheng Zhu

**Affiliations:** IRCCS Istituto Romagnolo per lo Studio dei Tumori (IRST) "Dino Amadori", Italy; ^1^Department of Urology, Xijing Hospital, Fourth Military Medical University, Xi’an 710032, Shaanxi, China; ^2^Department of Urology, First Affiliated Hospital of Kunming Medical University, Kunming 65000, Yunnan, China; ^3^College of Life Sciences, Northwest University, Xi’an 710068, Shaanxi, China; ^4^School of Clinical Medicine, Xi’an Medical University, Xi’an 710021, Shaanxi, China

**Keywords:** Bladder cancer, advanced urothelial carcinoma, targeted therapy, combination therapy, molecular alteration

## Abstract

Bladder cancer (BC) is the tenth most common malignancy globally. Urothelial carcinoma (UC) is a major type of BC, and advanced UC (aUC) is associated with poor clinical outcomes and limited survival rates. Current options for aUC treatment mainly include chemotherapy and immunotherapy. These options have moderate efficacy and modest impact on overall survival and thus highlight the need for novel therapeutic approaches. aUC patients harbor a high tumor mutation burden and abundant molecular alterations, which are the basis for targeted therapies. Erdafitinib is currently the only Food and Drug Administration (FDA)-approved targeted therapy for aUC. Many potential targeted therapeutics aiming at other molecular alterations are under investigation. This review summarizes the current understanding of molecular alterations associated with aUC targeted therapy. It also comprehensively discusses the related interventions for treatment in clinical research and the potential of using novel targeted drugs in combination therapy.

## Introduction

In 2020, bladder cancer (BC) was declared the tenth most frequently diagnosed cancer globally, with an estimated 573,000 new cases, of which 213,000 patients died [1]. BC originates from the transitional epithelium, and urothelial carcinoma (UC) is its predominant subtype, representing over 90% of all cases. Based on the invasion depth, BC is categorized as non-muscle-invasive BC (NMIBC) and muscle-invasive BC (MIBC). Approximately 75% of BC patients are NMIBC at diagnosis [2]. MIBC involves tumors invading the muscular layer of the bladder wall that have an elevated tendency to spread to lymph nodes and other organs [3]. Notably, the 5-year survival rate for patients with locally advanced UC (aUC) decreases from 70% to 38% compared with that for patients with localized cancer. By contrast, the 5-year survival rate for metastatic UC (mUC) is only 6%. These two conditions are collectively referred to as aUC [4].

**Figure 1:**
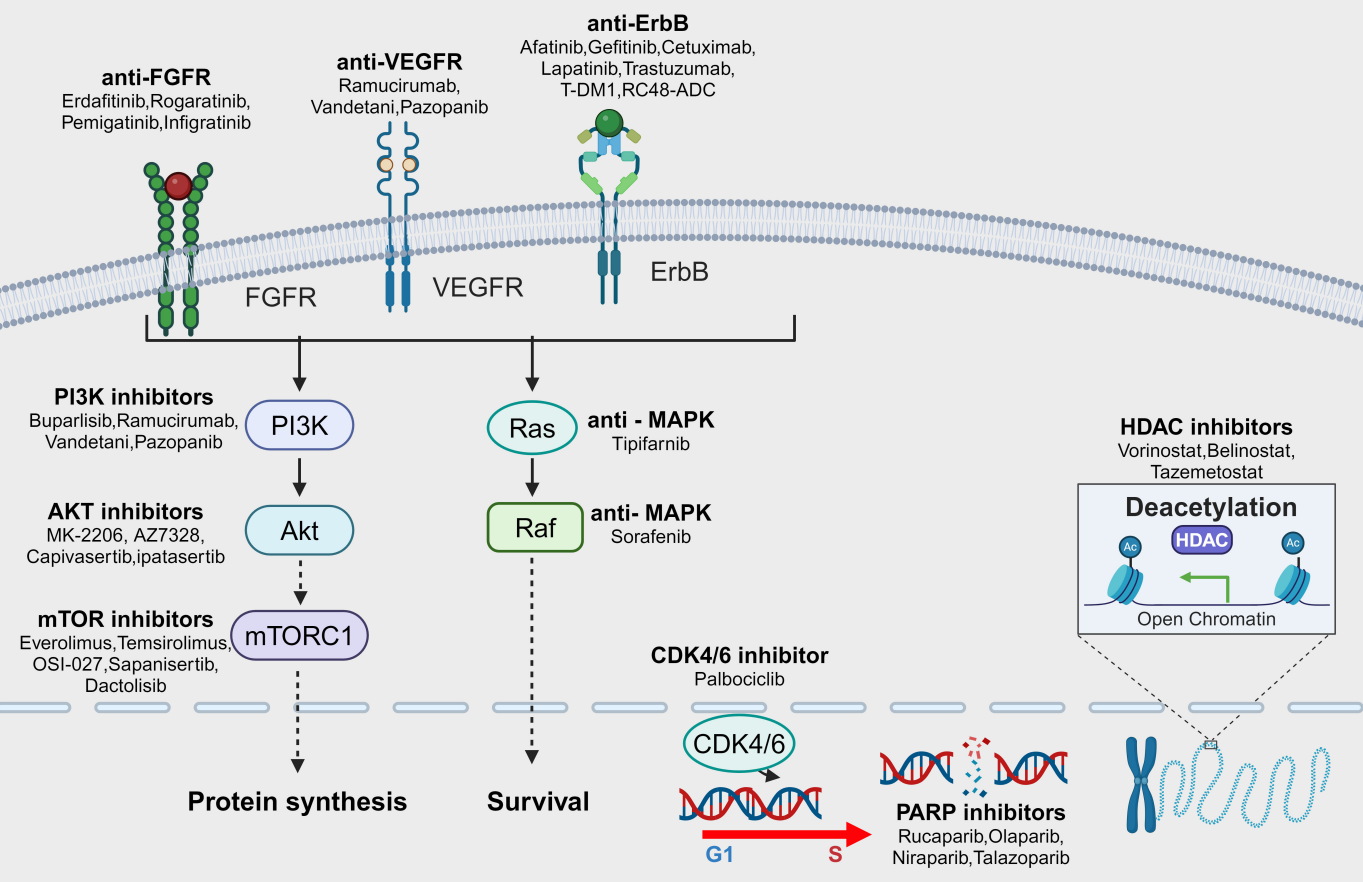
Pathways of targetable mutations in advanced urothelial carcinoma (aUC) and corresponding targeted drugs. The common mutation pathways in aUC are receptor tyrosine kinases (RTKs), such as fibroblast growth factor receptor (FGFR), erythroblastic oncogene B (ErbB), and vascular endothelial growth factor receptor (VEGFR). In addition, intracellular signaling pathways include phosphoinositide 3-kinase (PI3K)/protein kinase B (Akt)/mammalian target of rapamycin (mTOR) pathway, rat sarcoma (RAS)-mitogen-activated protein kinase (MAPK) pathway, DDR, and cell cycle regulation. Mutations in these pathways are closely related to the development of UC, and corresponding targeted drugs have already appeared and their effects have been investigated in clinical studies. mTORC1: mTOR complex 1; RAF: rapidly accelerated fibrosarcoma; T-DM1: trastuzumab-emtansine; RC48-ADC: disitamab vedotin; G1: gap phase 1; S: synthesis phase; CDK4/6: cyclin-dependent kinases 4/6; PARP: poly ADP-ribose polymerase; HDAC: histone deacetylase. Created with Biorender.com (accessed on April 28, 2024)

The first-line therapy for aUC patients is cisplatin-based chemotherapy. However, only approximately 30–50% of UC patients are eligible for first-line cisplatin-based chemotherapy, and the median overall survival (OS) in aUC patients is approximately 15 months [5, 6]. Although immunotherapies, including immune checkpoint inhibitors (ICIs) and antibody-drug conjugates (ADCs), are crucial additions to the therapeutic arsenal, additional therapeutic options are still required [6–9]. The Cancer Genome Atlas (TCGA) initiated aUC-specific program to discover genomic alterations and investigate their availability for targeted therapies [7]. Extensive studies on the molecular characterization of UC have underscored the crucial role of receptor tyrosine kinases (RTKs) in tumor development. Many classical signaling pathways are altered in UC, with the RTK/rat sarcoma (RAS)/phosphatidylinositol 3-kinase (PI3K) pathway being altered in 71% of cases. Alterations in cell cycle regulation and DNA damage response (DDR) are also common [8]. These altered classical signaling pathways allow us to use targeted therapeutic interventions. Current studies on drugs specifically targeting these pathways are shown in Figure 1. In this review, we describe the ongoing development of targeted therapies and their combinations in aUC, including their benefits and adverse effects. We also discuss possible future directions for clinical applications of such agents.

## Targeted therapies aiming RTKs

Kinase mutations are linked to UC [9]. RTKs, a predominant tyrosine kinases (TK) subtype, can phosphorylate tyrosine residues on substrate proteins. RTK has a protein structure similar to that of TK with an extracellular ligand-binding domain, a transmembrane helix, and an intracellular region that includes a juxtamembrane regulatory region, the TK domain (TKD), and a carboxy (C) end tail. RTKs mediate intercellular communication and regulate multiple biological functions, including cellular growth, motility, differentiation, and metabolism [10]. RTKs are upregulated in different tumor types and are considered oncogenes associated with cancer development and progression [11]. Therefore, targeting RTKs and suppressing their expression may generate positive outcomes in a clinical setting.

### Fibroblast growth factor receptor inhibitors

The fibroblast growth factor receptor (FGFR) is regarded as a promising locus for targeted therapy in UC. The FGFR family is composed of 4 transmembrane receptors (FGFR1–4). The structural domain spanning the cell membrane connects the extracellular and cytoplasmic regions of the cell. This domain comprises approximately 20 non-conserved hydrophobic amino acids, followed by basic residues. These components of the domain facilitate the attachment of the receptor to the membrane, thereby leading to translocation. TKDs and tyrosine phosphorylation sites are present within the intracellular region, whereas the juxtamembrane region of FGFR facilitates receptor dimerization [12]. Fibroblast growth factors (FGF) bind to the inactive monomeric conformation of FGFR and undergo conformational changes that promote dimerization and trans-phosphorylation of the structural domain of the intracellular TK. Activated FGFRs trigger downstream signaling through various pathways [13]. The FGF/FGFR physiological signaling axis is pivotal in organ development and metabolism. Broad-spectrum tumorigenesis that facilitates tumor growth, proliferation, differentiation, and survival is associated with dysregulation of this axis.

Helsten et al. [14] reported the presence of *FGFR* variants in multiple human cancers. These variants were reported in approximately 7.1% of the nearly 4,000 analyzed tumors. Specifically, UC involved approximately 15% of cases with somatic *FGFR3* mutations, approximately 7% with *FGFR1* amplification, and approximately 6% with gene fusions. *FGFR* fusions are of two types. Type 1 fusions, commonly observed in hematological tumors, involve chromosomal translocations affecting the structural domains of kinases. Type 2 fusions mostly cause rearrangements in solid tumors, thereby forming chimeric transmembrane FGFRs [15]. Younger, non-smoking Asian patients are more prone to the *FGFR3-*transforming acidic coiled-coil containing protein 3 (*TACC3*) gene fusion, and this fusion is associated with MIBC [16]. *TACC3* is involved in stabilizing and organizing the mitotic spindle [17]. When the *FGFR3-TACC3* fusion is formed, the TACC coiled-coil structural domain persistently phosphorylates crucial tyrosine residues. This fusion event improves downstream signaling pathways, including mitogen-activated protein kinase (MAPK), PI3K/protein kinase B (Akt), and signal transducer and activator of transcription 3 (STAT3) [18, 19]. Preclinical studies on the *FGFR3-TACC3* fusion-harboring cell line observed an enhancement in cell proliferation. Furthermore, *in vivo* studies conducted on nude mice evidenced that this fusion augments the activation of downstream proteins, including extracellular signal-regulated kinase (ERK) and STAT3 [20]. The *FGFR3-TACC3* fusion also leads to an altered *TACC3* function, potentially inducing mitotic defects and aneuploidy [21].

*FGFR3* dysregulation through mutation, overexpression, or both is observed in 54% of aUC cases [22]. Of note, the luminal-papillary urothelial subtype was recently found to possess a high frequency of *FGFR3* genomic alterations (65.2%) [7, 8]. Moreover, *FGFR3* mutations are associated with 5–20% of MIBC [22, 23]. Typically, ligand-dependent dimerization, activation, and signaling are actuated by aberrations that incorporate point mutations in extracellular regions [24]. Aggressive tumors exhibit overexpression of wild-type *FGFR3*, which induces ligand-dependent dimerization and activation [24].

*FGFR1* genomic aberrations have received relatively less attention than *FGFR3* genomic aberrations, but they are reported to be prevalent in 7–14% of cases [25]. FGFR1 promotes tumor proliferation and survival by activating MAPK and inducing epithelial-to-mesenchymal transition [26].

#### Erdafitinib

Erdafitinib (JNJ42756493), targeting the RTK family of FGF, is the first FDA-approved targeted therapy for aUC patients who have received platinum-based chemotherapy and have activating *FGFR2* or *FGFR3* mutations or fusions [27]. Preclinical studies have demonstrated the FGFR selectivity of erdafitinib, specifically over other kinases such as the vascular endothelial growth factor receptor (VEGFR). Additionally, in FGFR-altered cell lines, FGFR, FGFR substrate 2 (FRS2), phospholipase C γ1 (PLCγ1), and ERK1/2 phosphorylation levels are consistently reduced by deficient nanomolar erdafitinib concentrations, which results in the inhibition of proliferation of various FGFR-positive tumor cell lines [27].

The trial BCL2001 with 99 aUC participants assessed the efficacy and safety of erdafitinib [28, 29]. The first participant group had one or more *FGFR3* mutations (*n* = 74), while the other group had *FGFR2/3* fusions (*n* = 25). All study participants had undergone disease progression after platinum-based chemotherapy [29]. The findings revealed that 40% of the patients achieved an objective response rate (ORR), 3% achieved a complete response (CR), and 37% achieved a partial response (PR). Patients with *FGFR2/3* fusions had an ORR of 16%, whereas those with *FGFR3* mutations had a higher ORR of 49%. A 1-year OS rate of 49% and OS for 11.3 months [95% confidence interval (CI): 9.7–15.2] were secondary endpoints of the trial [28]. All patients developed at least one adverse event (AE) in the trial, with 67% of AEs being classified as grade 3 or 4 and hyperphosphatemia being the most common AE. Other more prevalent AEs were stomatitis, diarrhea, and dry mouth [29]. A study involved a median efficacy follow-up of approximately 2 years in 101 aUC patients with prespecified FGFR alterations. Treatment with selected erdafitinib regimens demonstrated a manageable safety profile and stable activity [28].

In the THOR study (NCT03390504), an ongoing phase III open-label, randomized, multicenter trial, the effectiveness and security of erdafitinib are evaluated [30]. The trial participants were divided into two cohorts based on the prior therapy they had received: cohort 1 comprised patients who had received the programmed cell death ligand 1 (PD-L1) inhibitor medication, while cohort 2 included patients who had undergone treatment without the PD-L1 inhibitor. The cohort 1 patients were randomly assigned in a 1:1 ratio to receive erdafitinib or chemotherapy (docetaxel or vinflunine). The cohort 2 patients were randomized to receive erdafitinib or pembrolizumab in a 1:1 ratio. The study’s primary endpoints were OS, progression-free survival (PFS), ORR, duration of remission (DOR), and patient-reported outcomes. The secondary endpoints were safety and pharmacokinetics. Data from the 266 patients were published as results of the interim analysis of cohort 1 in the THOR study, with 136 assigned to receive receiving erdafitinib and 130 randomized to receive chemotherapy [31]. The analysis revealed that the erdafitinib-treated patients achieved a median OS of 12.1 months at a median follow-up of 15.9 months. While patients on chemotherapy had a median OS of 7.8 months with a hazard ratio (HR) of 0.64 [95% CI: 0.47–0.88; *P* = 0.005]. Compared with chemotherapy, erdafitinib significantly improved median PFS (5.6 months *vs.* 2.7 months) and ORR (46% *vs.* 12%) [31]. The analysis revealed a consistent OS benefit of erdafitinib compared with chemotherapy across various subgroups. These subgroups were classified according to factors such as the baseline Eastern Cooperative Oncology Group performance status, prior treatment regimen, presence of visceral metastasis, primary tumor location, type of genetic alteration, and type of chemotherapy [31]. These findings evidenced the use of erdafitinib as a treatment for patients with FGFR-altered aUC after PD-L1 therapy. Through RNA sequencing and *in vitro* experiments, Xing et al. [32] identified the role of prolyl 4-hydroxylase subunit α2 (P4HA2) in conferring UC resistance to erdafitinib. P4HA2 interacts with hypoxia-inducible factor 1-alpha to form a positive feedback loop, thereby reducing intracellular reactive oxygen species levels, and has a key role in acquired resistance development, which suggests that P4HA2 can potentially be a target for UC management [32, 33].

### Other selective FGFR inhibitors

In a phase I study, rogaratinib (BAY1163877), a pan-FGFR inhibitor, exhibited promising efficacy and safety in aUC patients with FGFR1–3 mRNA overexpression [34, 35]. A phase II/III study (NCT03410693) investigated the effectiveness and safety of rogaratinib in FGFR mRNA-positive aUC patients who had received a platinum-based regimen [36]. The patients were assigned randomly in a 1:1 ratio to receive rogaratinib (*n* = 87) or chemotherapy (*n* = 88). In this trial, OS was the primary outcome. The interim analysis suggested that the combination of FGFR-targeted therapy with chemotherapy resulted in comparable effectiveness and a well-tolerated safety profile in FGFR-altered UC patients. According to exploratory trials, FGFR3 DNA alterations associated with FGFR1–3 mRNA overexpression may be a more reliable predictor of response to rogaratinib [36].

Pemigatinib (INCB054828) is an ATP-competitive inhibitor selectively and reversibly targeting FGFR1–3. Pemigatinib significantly inhibited FGFR1, 2, and 3 at half-maximal inhibitory concentrations (IC50) of 0.4 nmol/L, 0.5 nmol/L, and 1.0 nmol/L, respectively. However, its activity against FGFR4 was comparatively lower, with its IC50 being 30 nmol/L [37]. In preclinical investigations, pemigatinib demonstrated promising antitumor activity by effectively inhibiting the growth of FGFR-overexpressing tumor cell lines [37]. In the FIGHT-101 study, pemigatinib used against UC demonstrated a good tolerance and safety profile [38]. In that study, hyperphosphatemia was the most repeatedly observed treatment-related AE (TRAE) with an incidence of 75.0%. Conversely, fatigue was the most common grade ≥ 3 TRAE with an incidence of 10.2% [38]. The FIGHT-201 study (NCT02872714) is a completed phase II, open-label, multicenter study that evaluated the effectiveness and security of pemigatinib for aUC patients featuring FGF/FGFR alterations. Patients with FGFR3 mutations or fusions who received pemigatinib in an intermittent dose (ID, 2-weeks-on/1-week-off therapy) or a continuous dose (CD, no planned dose hold) were classified into cohort A-ID and cohort A-CD, respectively. Patients who had other FGF/FGFR alterations and received pemigatinib as an ID were assigned to B-ID. The published findings of this trial revealed that the ORR in cohort A-CD, including 101 patients was 17.8% (95% CI: 10.92–26.70%). In cohort A-ID, including 103 patients, the ORR was 23.3% (95% CI: 15.54–32.66%). Additionally, cohort B-ID, including 44 patients, had an ORR of 6.8% (95% CI: 1.43–18.66%). The combined cohort (*n* = 147) under the ID regimen (A-ID + B-ID) had an ORR of 18.4% (95% CI: 12.47–25.59%). The combined cohort (A-ID and A-CD, *n* = 204) included patients with FGFR3 mutations or fusions and exhibited an ORR of 20.6% (95% CI: 15.26–26.79%). Additionally, the combined cohort (*n* = 248), including patients with FGFR3 mutations or fusions in cohort A and those with other FGF/FGFR alterations in cohort B, had an ORR of 18.1% (95% CI: 13.55–23.52%). The PFS was 4.27 months (95% CI: 3.91–6.05) and 4.04 months (95% CI: 3.45–4.17) in the A-ID and A-CD groups, respectively, while that in the B-ID group was 2.04 months (95% CI: 1.87–2.17). The incidences of serious AEs in cohorts A-ID, B-ID, and A-CD were 43.69%, 59.09%, and 47.52%, respectively. The other AEs (excluding serious) observed in the cohorts were weakness, fatigue, hyperphosphatemia, alopecia, dry eye, abdominal pain, constipation, diarrhea, dry mouth, nausea, stomatitis, urinary tract infection, and taste disorders.

Infigratinib (BGJ398) is an oral FGFR1–3 inhibitor with selectivity. In preclinical trials, infigratinib exhibited a dose-dependent ability to reduce tumor growth in UC xenograft models. Additionally, it effectively lowered phosphorylated FRS2 (pFRS2) and phosphorylated MAPK (pMAPK) levels, thus displaying significant antitumor activity [39]. A phase I study revealed that infigratinib has a tolerable safety and exerts antitumor effects in FGFR3-altered UC patients [40]. Of the 67 infigratinib-treated patients who were ineligible for platinum-based chemotherapy, the remission rate was 25.4% and the disease control rate (DCR) was 64.2%. The chief treatment-emergent toxicities in these patients were hyperphosphatemia, elevated creatinine levels, fatigue, and constipation [41]. In a retrospective study analyzing 67 infigratinib-treated patients, the response rate in patients with hyperphosphatemia was as high as 33.3% (95% CI: 20.4–48.4), whereas that in patients without hyperphosphatemia was only 5.3% (95% CI: 0.1–26.0). Additionally, the DCR was also higher in patients with hyperphosphatemia (75.0%, 95% CI: 60.4–86.4) than in those without hyperphosphatemia (36.8%, 95% CI: 16.3–61.6). These findings suggest that elevated blood phosphorus levels and improved clinical outcomes are potentially correlated [42]. However, larger cohort studies and prospective trials are warranted to further validate these observations and to assess the clinical significance of hyperphosphatemia as a biomarker. The PROOF 302 trial (NCT 04197986) is currently being conducted to address this issue [43]. Although the sponsor terminated the study prematurely, genomic analysis and evaluations of correlations to primary and secondary endpoints are ongoing. The ongoing genomic analysis offers valuable insights into the frequency of FGFR3 alterations [43].

Futibatinib (TAS120) is an orally administered novel inhibitor potent, selective, and irreversible against FGFR1–4. In preclinical studies, futibatinib displayed the potential to inhibited UC growth effectively [44]. Furthermore, a phase I trial involving 19 UC patients evidenced the clinical activity and tolerability of futibatinib during bladder tumor treatment [45]. The trial reported an ORR of 15.8% (95% CI: 3.4–39.6%), including 3 cases achieving a PR and 6 cases of stable disease (SD). The DCR was 47.4%, and the DCR, including SD lasting for > 16 weeks, was 20.5%. Importantly, these results were observed in patients who had received multiple lines of treatment, with > 50% of the enrolled patients having undergone more than three lines of therapy [45]. Hyperphosphatemia was the primary reason for the dose adjustment of futibatinib in two studies (NCT02052778, JapicCTI-142552) on patients administered with 20 mg/day futibatinib [46]. Of note, 85% of patients with hyperphosphatemia received phosphate-binding agents, whereas 30% received phosphate-solubilizing agents. However, no significant difference was noted in response time. Additionally, analgesics (55% of patients with nail lesions; 71% of patients with palmoplantar erythrodysesthesia) and corticosteroids (37% of patients with rash) were the commonly administered concomitant medications. Overall, based on the analysis, futibatinib can be concluded to demonstrate a consistent and manageable safety profile [46].

#### FGFR inhibitors in combination therapies

##### Combinations with ICIs

**Figure 2:**
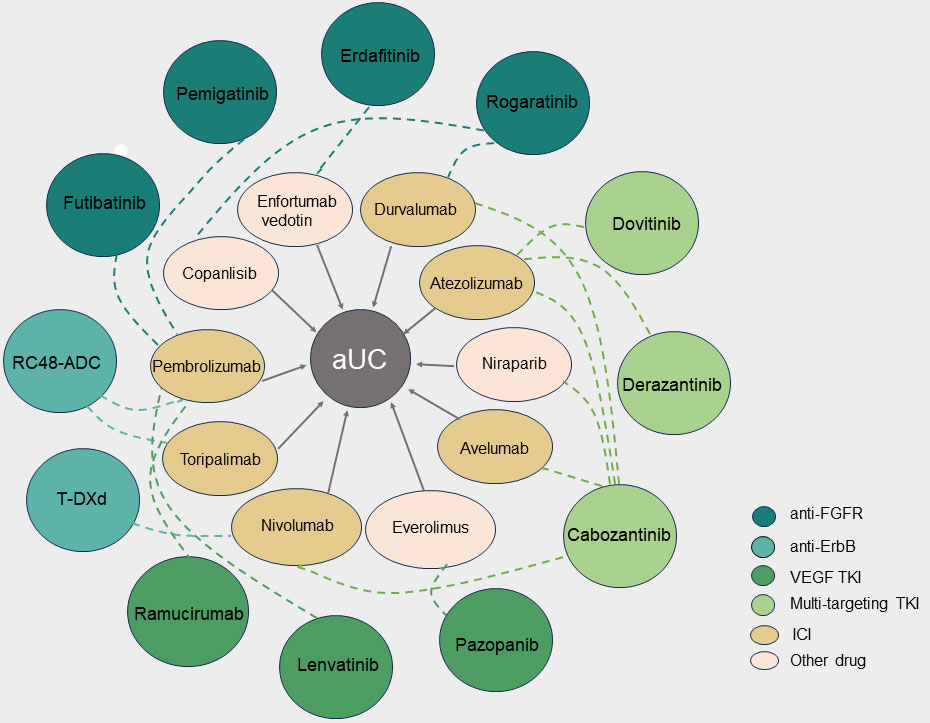
Combination therapies with targeted tyrosine kinase inhibitors (TKI) in advanced urothelial carcinoma (aUC). The different colored circles on the outside represent TKI against different targets, and the ellipses on the inside represent another drug in the combination, with the dotted line indicating the relationship between the combination of two drugs for aUC. FGFR: fibroblast growth factor receptor; VEGF: vascular endothelial growth factor; ICI: immune checkpoint inhibitor; RC48-ADC: disitamab vedotin; T-DXd: trastuzumab deruxtecan; ErbB: erythroblastic oncogene B

A phase Ib study (NCT04963153) supported by the National Cancer Institute (NCI) is currently in the recruitment phase. This study intends to evaluate the feasibility and security of using erdafitinib and enfuzumab (E/V) in a combination for aUC patients with genomic alterations in FGFR2/3 activation (Figure 2). After treatment with chemotherapy and ICIs, these patients experienced disease progression. Determining the maximum tolerated dose of the E/V combination and the recommended phase II dose (RP2D) are the major study goals [47].

The Norse study revealed that the combination of erdafitinib and cetrelimab (CET) exhibited both clinical efficacy and tolerability as the first-line treatment for aUC patients non-eligible for cisplatin therapy. The median follow-up period was 14.2 months. The erdafitinib + CET group (*n* = 44) had an ORR of 54.5%, with 6 patients (13.6%) achieving a CR. The 12-month OS rate was 68%. Conversely, the erdafitinib monotherapy group (*n* = 43) had an ORR of 44.2%, and its 12-month OS rate was 56%. The most commonly reported AEs of any grade in the erdafitinib + CET and erdafitinib monotherapy groups were hyperphosphatemia (68.9% *vs.* 83.7%), stomatitis (59.1% *vs.* 72.1%), and diarrhea (45.5% *vs.* 48.8%). TRAEs of grade ≥ 3 were observed in 45.5% and 46.5% of patients in the erdafitinib + CET and erdafitinib monotherapy groups, respectively. One patient in the erdafitinib + CET group had a CET-related fatality attributable to lung failure. Overall, the safety profile of the erdafitinib and CET combination therapy is consistent with the known safety profiles of individual treatments with both drugs [48].

Another ongoing phase II trial (NCT05564416) is recruiting localized UC patients non-eligible for chemotherapy. The trial’s goal is to determine the effectiveness of erdafitinib in combination with atezolizumab or as a monotherapy (Table 1).

**Table 1:** Ongoing clinical trials of FGFR-targeted therapy in UC

**Drugs**	**Targets**	**Combination**	**Conditions**	**Phase**	**NCT**
Erdafitinib	FGFR2/3	Null	Rec UC | *FGFR3* gene mutation	II	NCT04917809
Erdafitinib	FGFR2/3	Null	UC	I	NCT05316155
Erdafitinib	FGFR2/3	Null	UC	I	NCT05567185
Erdafitinib	FGFR2/3	Gemcitabine + mitomycin C	UC	II	NCT04172675
Erdafitinib	FGFR2/3	Atezolizumab	UC | MIBC	II	NCT05564416
Erdafitinib	FGFR2/3	Enfortumab vedotin	Adv UC	I	NCT04963153
Rogaratinib	FGFR1–3	Null	UC	II	NCT04040725
Rogaratinib	FGFR 1-3	Atezolizumab	UC	I	NCT03473756
Pemigatinib	FGFR1–3	Null	Rec UC | NMIBC	II	NCT03914794
Infigratinib	FGFR1–3	Null	Solid Tumor	II	NCT05019794
Infigratinib	FGFR1–3	Null	Adv/Met malignant solid neoplasm	II	NCT04233567
Infigratinib	FGFR1–3	Null	Adv solid tumor	I/II	NCT05222165
Infigratinib	FGFR1–3	Null	UC | NMIBC	Not applicable	NCT02657486
Futibatinib	FGFR1–4	Null	UC	I/II	NCT02052778
ICP-192	FGFR 1–4	Null	UC	II	NCT04492293
ICP-192	FGFR 1–4	Null	UC	I/II	NCT04565275
TYRA-300	FGFR3	Null	UC	I	NCT05544552

Null in Combination indicates that the trial is monotherapy. FGFR: fibroblast growth factor receptor; UC: urothelial carcinoma; MIBC: muscle-invasive bladder cancer; NMIBC: non-MIBC; Adv: advanced; Met: metastatic; Rec: recurrent; NCT: national clinical trial; ICP-192: gunagratinib; |: and

The phase I b/II trial FORT-2 investigated the rogaratinib and atezolizumab combination in aUC patients non-eligible for cisplatin therapy and displayed *FGFR* overexpression [49]. The preliminary results of the study demonstrated a DCR of 83%. In mUC patients, who had high FGFR1/3 mRNA expression and generally low/negative PD-L1 expression, the combination therapy resulted in favorable clinical efficacy and tolerability. Another ongoing phase I study (NCT03517956), known as ROCOCO, evaluates the combined use of rogaratinib and copanlisib in patients with solid tumors and FGFR positivity.

A randomized, open-label phase II clinical trial, FIGHT-205 study (NCT04003610), compares the effectiveness and safety of the combination of pemigatinib and pembrolizumab with pemigatinib alone, as well as standard-of-care treatments such as carboplatin + gemcitabine and pemigatinib + pembrolizumab. This trial specifically focuses on aUC patients non-eligible for cisplatin-based therapy but have an FGFR3 mutation or rearrangement. Unfortunately, this study was terminated because of business decisions.

The experimental drug, futibatinib, is currently being evaluated in an open-label phase II study (NCT04601857) [50]. This study investigated futibatinib in combination with pembrolizumab among aUC patients who have received platinum-based treatment [50]. The preliminary safety results demonstrated that the combination therapy is tolerable in this specific patient population [50].

##### Combination with targeted therapy

In an ongoing phase I clinical trial, the ROCOCO study (NCT03517956), the combination of rogaratinib and copanlisib is being evaluated in patients with FGFR-positive advanced solid tumors.

A recent study reported the synergistic effects of FGFR inhibitors and histone deacetylase (HDAC) inhibitors both *in vitro* and *in vivo*, specifically targeting UC patients with FGFR3 fusion [51]. Quisinostat, an HDAC inhibitor, can downregulate FGFR3 expression by inhibiting its translation process. Additionally, quisinostat can sensitize UC cells to erdafitinib by downregulating the hepatocellular carcinoma-derived growth factor [51].

### Erythroblastic oncogene B receptors inhibitors

Epidermal growth factor receptor [EGFR, also known as erythroblastic oncogene B-1 (ErbB-1)or human epidermal growth factor receptor 1 (HER1)], ErbB-2 (HER2/neu), ErbB-3 (HER3), and ErbB-4 (HER4) are the four receptors of the ErbB family. By activating downstream pathways such as MAPK and PI3K/Akt, these cell membrane-bound RTKs become a crucial player in cell proliferation [52–55]. EGFR, ErbB-2, and ErbB-3 mutations or amplifications have been reported in MIBC. EGFR aberrations account for approximately 6–14% of MIBC cases, ErbB-2 mutations for 6–23% of cases, and ErbB-3 mutations for 6% of MIBC cases [8, 56, 57]. Because of the prevalence of these genetic aberrations, the ErbB family has become a promising target for anticancer drug development.

#### ErbB-1 receptor inhibitors

Afatinib is a potent and specific inhibitor that irreversibly targets both EGFR and HER2 and has produced encouraging outcomes in preclinical investigations. These studies have highlighted that afatinib can impede UC cell proliferation and invasion by selectively modulating the EGFR signaling pathway and promoting apoptosis [58]. In a phase II study, afatinib was administered to patients with platinum-refractory aUC [59]. Among the 21 analyzed tumors, 83.3% of patients (5 out of 6) with HER2 and/or ErbB-3 alterations remarkably attained an average PFS of 3 months (range: 5.0–10.3 months). By contrast, none of the 15 unaltered patients achieved the 3-month PFS (*P* < 0.001). The median time to worsening/discontinuation was 6.6 months for the HER2/ErbB-3 variant-harboring patients compared with 1.4 months for those without the variant (*P* < 0.001) [59]. Thus, afatinib can effectively prolong the survival of patients with platinum-refractory UC exhibiting HER3 or ErbB-3 alterations [59].

Dacomitinib, like afatinib, is a second-generation EGFR TK inhibitor (TKI). Preclinical studies have unveiled the antitumor properties of dacomitinib and its potential to exert synergistic effects with radiation therapy. However, the outcomes of clinical trials have overall been less favorable [60, 61].

In preclinical UC models, gefitinib, an orally active EGFR-TKI, exhibited promising therapeutic potential [62, 63]. A phase II study assessed the effectiveness of chemotherapy combined with gefitinib in aUC patients. However, the aforementioned combination had no significant impact on patient progression [64].

Cetuximab, an EGFR-TKI inhibitor, effectively suppressed angiogenesis and displayed antitumor effects in bladder migratory cell carcinoma grown in nude mice. The antitumor activity of cetuximab was boosted by paclitaxel [65–67]. However, a phase II study reported no improvement in patient outcomes with cetuximab, but an increase in AEs [68]. Interestingly, the phase I/II trial (TYXEDO) concluded that cetuximab addition to radiochemotherapy is feasible and safe [69].

Erlotinib has shown potential as a treatment option for UC. A phase II study reported the beneficial clinicopathological efficacy of the neoadjuvant use of erlotinib in patients undergoing radical cystectomy (RC) for invasive UC [70]. Additionally, this study found that the combined pulsed or intermittent administration of erlotinib and naproxen significantly hindered UC growth [71].

#### ErbB-2 receptor inhibitors

##### Trastuzumab and derivants

Trastuzumab is an ErbB-2-targeting monoclonal antibody. In a multicenter phase II trial conducted in 2007, the combination of trastuzumab, paclitaxel, carboplatin, and gemcitabine produced promising results. Among the 44 HER2-positive patients included in the trial, 31 (70%) patients achieved a PR or CR [72]. Oudard et al. [73] conducted a phase II trial to identify the true effect of trastuzumab on UC. The study determined the effectiveness of gemcitabine plus platinum, with or without trastuzumab, in aUC patients exhibiting high HER2 expression levels. Among the 563 patients, only 13.3% patients had HER2-positive tumors. Additionally, no apparent differences were noted in the ORR, median PFS, and median OS [73].

A case report showcased the effectiveness of trastuzumab and chemotherapy combination therapy for recurrent UC patients with *HER2* amplification. Patients, who had received 75 mg/m^2^ cisplatin every 3 weeks as a second-line treatment, also underwent 5 cycles of trastuzumab at 6 mg/kg (administered every 3 weeks) with an initial dose of 8 mg/kg. Subsequently, the patient achieved a clinical CR for up to 34 months [74]. Similarly, another aUC patient achieved complete tumor remission after receiving experimental third-line therapy with trastuzumab and gemcitabine. This patient had completed 8 cycles of treatment [75]. However, the optimal approach for selecting patients for this treatment combination remains uncertain.

Preclinical studies and a phase II trial have demonstrated that ErbB-2-targeted therapies involving trastuzumab or trastuzumab-emtansine (T-DM1) can significantly benefit patients. In T-DM1, trastuzumab is combined with a tubulin-binding agent, mertansine, by using a stable thioether linker [76]. The KAMELEON study unveiled that a specific subset of patients with HER2-positive UC showed positive responses to T-DM1 [77]. Unfortunately, the study was prematurely terminated because of challenges encountered in patient recruitment [77].

The ADC trastuzumab deruxtecan (T-DXd) is developed to specifically target HER2. It has been approved for managing selected HER2-expressing tumors. Currently, an open-label phase II study known as DP-02 (NCT04482309) is exploring the efficacy of 5.4 mg/kg T-DXd administered every 3 weeks to aUC patients (Table 2) [46, 78]. Patients who have progressed after receiving systemic therapy or those who have exhausted all other treatment options are eligible for this study. This study uses immunohistochemistry (IHC) testing to evaluate HER2 expression. IHC 3+ or IHC 2+ is determined through local or central testing. Within the subgroup of UC patients with IHC 3+ expression (*n* = 16), the ORR was 56.3%. In the UC patients with IHC 2+ expression (*n* = 20), the ORR was 35.0% [78]. These interim findings suggest that T-DXd can be a promising new treatment for HER2-expressing UC.

**Table 2:** Ongoing clinical trials of ErbB-targeted therapy in UC

**Drugs**	**Targets**	**Combination**	**Conditions**	**Phase**	**NCT**
Afatinib	ErbB-1/2 (EGFR/ HER2)	Null	Adv UC	II	NCT02122172
Afatinib	ErbB-1/2 (EGFR/ HER2)	Null	Adv solid tumors	II	NCT02465060
Cetuximab	ErbB-1 (EGFR)	TTX-080	Cancer	I	NCT04485013
Cetuximab	ErbB-1 (EGFR)	SNK01	UC	I/II	NCT04464967
Lapatinib	ErbB-1/2 (EGFR/ HER2)	Null	UC	II/III	NCT00949455
Lapatinib	ErbB-1/2 (EGFR/ HER2)	Paclitaxel	UC	II	NCT01700010
Trastuzumab deruxtecan	ErbB-2 (HER2)	Null	UC	II	NCT04482309
Trastuzumab deruxtecan	ErbB-2 (HER2)	Nivolumab	UC	I	NCT03523572
Trastuzumab deruxtecan	ErbB-2 (HER2)	Pyrotinib	Met/Adv UC, HER2 positive	II	NCT05318339
Trastuzumab deruxtecan	ErbB-2 (HER2)	Tucatinib	Urologic neoplasms	II	NCT04579380
Trastuzumab deruxtecan	ErbB-2 (HER2)	AZD5305	UC	I/II	NCT04644068
RC48-ADC	ErbB-2 (HER2)	Null	UC | NMIBC	II	NCT05996952
RC48-ADC	ErbB-2 (HER2)	Toripalimab	UC | MIBC	II	NCT05297552
RC48-ADC	ErbB-2 (HER2)	Triplizumab	UC | MIBC, HER2 positive	II	NCT05356351
RC48-ADC	ErbB-2 (HER2)	Triplizumab	UC	II	NCT05016973
RC48-ADC	ErbB-2 (HER2)	Toripalimab	UC | MIBC, HER2 positive	II	NCT05979740
RC48-ADC	ErbB-2 (HER2)	pembrolizumab	UC	II	NCT04879329
RC48-ADC	ErbB-2 (HER2)	pembrolizumab	UC	III	NCT05911295
Trastuzumab emtansine	ErbB-2 (HER2)	Null	UC	II	NCT02675829

Null in combination indicates that the trial is monotherapy. ErbB: erythroblastic oncogene B; EGFR: epidermal growth factor receptor; HER2: human epidermal growth factor receptor 2; MIBC: muscle-invasive bladder cancer; NMIBC: non-MIBC; UC: urothelial carcinoma; Adv: advanced, Met: metastatic, Rec: recurrent; RC48-ADC: disitamab vedotin; NCT: national clinical trial

##### Other ErbB-2 receptor inhibitors

A phase III trial evaluated the effectiveness of lapatinib, a dual TKI targeting both EGFR and ErbB-2, in chemotherapy-treated aUC patients who presented with progressive disease. Patients exhibiting ErbB-1 or ErbB-2 protein expression were enrolled. The patients were randomized to receive lapatinib or a placebo. Unfortunately, the results of this were disappointing as no significant improvement in patient outcomes was observed among the lapatinib-treated patients [79]. Notably, on investigating lapatinib as an initial therapy for muscle-invasive UC in dogs, a distinct study found encouraging outcomes, including durable response rates, improved survival, and favorable tolerability. These findings support the potential use of lapatinib in canine patients with aUC [80].

Disitamab vedotin (RC48-ADC), is a humanized anti-ErbB-2 antibody linked to monomethyl auristatin E through a cleavable linker. During the phase I study, RC48-ADC produced positive safety results and displayed promising activities against solid tumors, thereby highlighting the effectiveness of RC48-ADC against HER2-positive cancers [81]. In a multicenter, open-label phase II study with 20 aUC patients exhibiting an HER2 expression status of IHC 3+ or 2+, RC48-ADC treatment resulted in an ORR of 51.2% (95% CI: 35.5–66.7%). The median PFS was 6.9 months (95% CI: 5.6–8.9), and the median OS was 13.9 months [95% CI: 9.1–not estimable (NE)]. Hypoesthesia, alopecia, and leukopenia were the most common TRAEs associated with RC48-ADC treatment. Of all the patients, 25 (58%) patients experienced grade 3 TRAEs, and no grade 4 or grade 5 TRAEs were reported. Thus, RC48-ADC exhibits good effectiveness and positive security in aUC patients who have received prior systemic chemotherapy [82].

#### ErbB receptor inhibitors in combination therapies

##### Combination with ICI

The RC48-C014 study evaluated the combination of RC48-ADC and toripalimab, an anti-programmed cell death protein 1 (PD-1) antibody, in aUC patients regardless of their HER2 status [83, 84]. As of November 18, 2022, 41 aUC patients were enrolled in this study. The patients achieved an ORR of 73.2% (95% CI: 57.1–85.8) and a CR rate of 9.8%. The ORR for treatment-naïve patients was 76.0%. Among the patients assigned to different HER2 IHC subgroups, the ORRs were 83.3%, 64.3%, and 33.3% for the IHC 3+/2+, IHC 1+, and IHC 0 subgroups, respectively. The ORR was 61.5% in the PD-L1-positive subgroup compared with 78.6% in the PD-L1-negative subgroup. The median PFS was 9.2 months (95% CI: 5.7–10.3), and the two-year OS rate was 63.2%. In this study, the most frequent TRAEs observed were elevated glutamic/glutamic aminotransferase, peripheral sensory neuropathy, shortness of breath, elevated gamma-glutamyltransferase, hypertriglyceridemia, and loss of appetite. Among all patients, 43.9% of patients developed grade ≥ 3 TRAEs, and 56.1% of patients (*n* = 41) developed immune-related AEs, with 14.6% of them being classified as grade ≥ 3. According to the study results, the RC48-ADC and toripalimab combination exhibits promising effectiveness and positive safety in aUC patients [84]. A case report demonstrates the effectiveness of RC48-ADC + pembrolizumab treatment in a 68-year-old man with HER2-positive aUC. Despite prior treatment failures, the patient demonstrated rapid response and long-term PFS (> 12 months) without severe AEs. These findings strongly indicate that the RC48-ADC and pembrolizumab combination has remarkable efficacy and safety in patients with HER2-positive aUC, which suggests a promising therapeutic strategy against aUC that needs to be further explored [85].

In a phase Ib study, the combination of ADC T-DXd and nivolumab is being investigated for aUC patients who have received prior platinum-based chemotherapy and exhibited HER2 protein expression, as determined through IHC testing. The ORR of 30 patients with high HER2 expression (2+ or 3+) was 36.7%. Responses were noted in both HER2-expressing 3+ and some 1+ patients, but response activity was higher in the 3+ patients. However, 23.5% of patients developed interstitial lung disease/pneumonitis, with one case leading to death [86].

##### Combination with other therapies

*In vivo*, bladder treatment with metformin and gefitinib significantly suppressed UC growth in homozygous in situ mice. This drug combination exerted substantial antiproliferative and anticolony-forming effects, as well as inhibited UC cell lines by inducing apoptosis. Gefitinib effectively inhibited EGFR signaling, thereby inhibiting ERK and Akt phosphorylation. Additionally, metformin augmented the inhibitory effect and facilitated gefitinib-induced activation of the MAPK signaling pathway. When intravesically instilled, this drug combination holds great promise as an excellent therapeutic approach for UC [87, 88]. Additionally, phenelzine, a metformin analog, when administered alone or in combination with gefitinib, seems promising as an effective agent for UC treatment [89].

### Vascular endothelial growth factor pathway inhibitors

Angiogenesis is a physiological process exploited by malignant tissues to promote tumor development. Using this pathway, tumors stimulate new blood vessel formation, which in turn offers oxygen and nutrients critical for their continued proliferation [90, 91].

Vascular endothelial growth factor-A (VEGF-A), a member of the VEGF protein family, interacts with VEGFR2 on endothelial cells to promote neovascularization [92]. Elevated VEGF levels are usually associated with adverse outcomes in UC patients [93]. For instance, Kanayama et al. [94] identified a correlation between serum VEGF levels and neoplasm characteristics such as stage, grade, vascular invasion, and other factors, and an inverse correlation with disease-free survival [94].

Given the significance of the relationship between tumor progression and angiogenesis, new therapeutic approaches are being developed in this area, including anti-VEGF/VEGFR antibodies, TKIs, and other drugs.

#### VEGF/VEGFR inhibitors

Bevacizumab is a monoclonal antibody that has been humanized and can directly bind to and inhibit all VEGF-A isoforms. Numerous preclinical studies have reported the inhibitory impact of bevacizumab on UC cell proliferation [95]. The combination of gemcitabine, cisplatin, and bevacizumab exhibited favorable OS and antiangiogenic therapy-associated toxic responses in aUC patients in a phase II study [96]. However, a phase III trial CALGB 90601 revealed that bevacizumab added to the gemcitabine + cisplatin combination yielded no remarkable increase in OS among aUC patients [97].

Ramucirumab, a recombinant human monoclonal antibody, binds directly to the extracellular structural domain of VEGFR2, thereby inhibiting angiogenesis [98]. By enrolling 140 aUC patients who exhibited disease progression within 1 year of initial platinum-based therapy, a phase II trial compared the effectiveness and security of ramucirumab or icrucumab combined with docetaxel and docetaxel-only therapy. The patients were undergoing platinum agent-based therapy or experiencing disease progression within 12 months. Compared with the chemotherapy-only group, the ramucirumab group had a significantly longer PFS. The median PFS was 5.4 months (95% CI: 3.1–6.9 months) and 2.8 months (95% CI: 1.9–3.6 months) for the ramucirumab and chemotherapy-only groups, respectively. However, OS exhibited no significant increase [99]. A randomized, double-blind phase III RANGE study involving 530 aUC patients determined the security and effectiveness of the ramucirumab + docetaxel combination in comparison to the docetaxel + placebo combination [100]. The patients were insensitive or non-responsive to chemotherapy. The median PFS notably increased with ramucirumab compared with placebo [4.1 months (95% CI: 3.3–4.8) *vs.* 2.8 months (95% CI: 2.6–2.9); HR: 0.696 (95% CI: 0.573–0.845); *P* = 0.0002]. Median OS exhibited no significant difference between the ramucirumab group [9.4 months (95% CI: 7.9–11.4)] and the placebo group [7.9 months (95% CI: 7.0–9.3); stratified HR: 0.887 (95% CI: 0.724–1.086); *P* = 0.25]. Additional follow-up data revealed that the ramucirumab and docetaxel combination offers substantial benefits in terms of PFS in platinum-refractory aUC patients; however, this therapy caused no statistically significant increase in OS [100].

The oral TKI vandetanib specifically targets VEGFR2 and EGFR. In preclinical models, vandetanib exerted synergistic effects with cytotoxic chemotherapy, especially cisplatin, by dose-dependently sensitizing UC cells [101]. Nevertheless, vandetanib incorporated into docetaxel-based treatment could not substantially increase PFS, ORR, or OS among the cohort of aUC patients who had undergone platinum-based chemotherapy in a double-blind randomized clinical trial [102]. Additionally, a phase II trial used the combination of vandetanib, carboplatin, and gemcitabine as a first-line treatment for aUC patients non-eligible for cisplatin. No substantiated evidence supported the improvement in clinical outcomes with vandetanib in this particular treatment context [103].

Pazopanib is an orally administered effective inhibitor. It selectively targets three VEGFRs, as well as the platelet-derived growth factor receptor (PDGFR), c-Kit, and FGFR TKs to exert its antiangiogenic effects [104]. A phase II clinical study of pazopanib for aUC patients reported an ORR of 17.1%, and all observed responses were PRs. The most common grade 3 TRAEs were hypertension, fatigue, and gastrointestinal and vaginal fistulas. Regrettably, 1 patient died due to a duodenal fistula associated with the tissue response to extensive tumor masses [105]. A phase II study with 19 aUC patients investigated pazopanib's role in aUC treatment [106]. Unfortunately, the trial was terminated as no significant therapeutic activity was observed [106]. Furthermore, pazopanib combined with vinflunine for aUC treatment resulted in poor tolerability [107]. However, the combination of paclitaxel and pazopanib produced a promising ORR of 54% among previously treated aUC patients [108].

#### VEGF inhibitors in combination therapies

A phase Ib multicohort study assessed the effectiveness of the ramucirumab + pembrolizumab combination in UC patients who had undergone platinum-based systemic therapy and developed disease progression. In the aUC patient cohort comprising 24 individuals, 3 patients exhibited a positive objective response, yielding an ORR of 13% (95% CI: 2.7–32.4%). The study thus suggested that the ramucirumab + pembrolizumab combination is tolerated well by UC patients and has notable objective antitumor activity. These findings imply a potential therapeutic benefit of this combination for this specific patient population [109].

The NCT02501096 study reported the outcomes of dosimetry and initial phase II extension of a phase Ib/II clinical trial that assessed the lenvatinib + pembrolizumab combination. The patient cohort included carefully selected individuals with advanced solid tumors. A manageable safety profile and a promising antitumor effect were observed [110]. All patients were administered lenvatinib at the recommended dose of 20 mg/day in combination with 200 mg pembrolizumab every 3 weeks until the patient’s disease progressed or toxicity was untolerated in the phase II segment of the study. In the cohort, the UC patients presented an objective response at 24 weeks and the overall ORR was 25% (5 out of 20; 95% CI: 8.7–49.1%). The median DOR was not reached (95% CI: 6.5–NE), and the median PFS was 5.4 months (95% CI: 1.3–NE months) [110]. A phase III study (NCT03898180) investigated the effects of the pembrolizumab + lenvatinib combination and pembrolizumab alone on aUC patients. The median PFS and median OS in the pembrolizumab group were 4.0 and 12.9 months, respectively. By contrast, the median PFS and median OS were 4.5 months [HR: 0.90 (95% CI: 0.72–1.14)] and 11.8 months [HR: 1.14 (95% CI: 0.87–1.48)], respectively, in the combination group. More AEs reported in the pembrolizumab group than in the combination group. This study was terminated early relative to the planned time and suggested that pembrolizumab combined with lenvatinib was no more effective but was associated with more risk than pembrolizumab in the aUC patients [111]. Additional clinical trials of lenvatinib for UC treatment are ongoing (Table 3).

**Table 3:** Ongoing clinical trials of VEGF-targeted therapy in UC

**Drugs**	**Targets**	**Combinations**	**Conditions**	**Phase**	**NCT**
Bevacizumab	VEGF	MK-7684A	UC	II	NCT05007106
Bevacizumab	VEGF	Dasatinib	Met/Adv UC	I	NCT04164069
Lenvatinib	VEGFR1–3	GI-101	Met/Adv UC	I/II	NCT04977453
Lenvatinib	VEGFR1–3	MK-7684A	UC	II	NCT05007106
Ramucirumab	VEGFR2	TRK-950	UC	I	NCT03872947

VEGF: vascular endothelial growth factor; UC: urothelial carcinoma; VEGFR1–3: vascular endothelial growth factor receptor 1–3; Adv: advanced; Met: metastatic; MK-7684A: the combination of vibostolimab and pembrolizumab; GI: SIM0323

Bellmunt et al. [112] conducted a study in 23 UC patients, of which 19 patients had aUC. The patients received everolimus and pazopanib (E/P). The study reported an ORR of 21%, which consisted of 1 CR case and three PR cases. Additionally, 8 patients exhibited SD. The DOR, PFS, and OS were reported at 6.5, 3.6, and 9.1 months, respectively. Thus, E/P was found to be safe for aUC patients. Moreover, this treatment approach may produce clinical benefits in patients with specific mammalian target of rapamycin (mTOR) or FGFR pathway alterations [112].

### Multitargeting TKI

#### Multitargeting TKI

Dovitinib (TKI258) is a pan-TKI that primarily targets VEGFR and PDGFR. Additionally, it targets FGFR1–3, feline McDonough sarcoma like TK 3, the stem cell factor receptor, and colony-stimulating factor receptor 1 [113]. In a phase II clinical trial (NCT01732107), dovitinib was evaluated for its efficacy in patients with BCG-refractory UC displaying FGFR3 mutation or overexpression [114]. However, despite the high potency of dovitinib against FGFRs, no therapeutic improvement was noted and the study was terminated [114].

Derazantinib is a multitargeting TKI with activity against FGFR1–3, colony-stimulating factor receptor 1, and VEGFR2. In a phase I study assessing derazantinib, genetic alterations in FGFRs were detected in 22 of the 80 recruited patients with advanced solid tumors. The most common AEs were fatigue (49%), increased alanine aminotransferase (ALT) levels (30%), and diarrhea (23%) [115].

Anlotinib is a highly potent oral multitargeted TKI with a favorable safety profile and exhibits activity against VEGFR, FGFR, PDGFR, and c-kit. Anlotinib effectively inhibits UC cell proliferation, migration, and invasion by suppressing ERK1/2 and Akt phosphorylation, as well as VEGF-a expression. Moreover, it demonstrates efficacy superior to that of erdafitinib in the treatment of UC patients harboring FGFR3 fusion mutations [116].

Famitinib is a TKI that effectively targets multiple receptors, including c-kit, VEGFR2, and PDGFRβ. It inhibits other kinases, such as Fms-like TK 1/3, rearranged during transfection, and AXL/MER. To assess the potential of combining carelizumab and famitinib as a monotherapy or combined therapy for genitourinary (GU) or gynecologic cancers, a phase II study was conducted [117]. This study enrolled 36 aUC patients who had progressed after receiving platinum-based chemotherapy. The median duration from enrollment to data cut-off was 11.9 months (range: 6.1–28.5 months), the ORR was 30.6% (95% CI: 16.3–48.1%), and the median DOR was 6.3 months (95% CI: 2.1–not reached). Notably, UC patients (*n* = 18) had a higher ORR of 38.9% (95% CI: 17.3–64.3%) and a median PFS of 8.3 months (95% CI: 4.1–not reached). The median DOR and OS were not determined for this subgroup, but the lower 95% CIs were 4.2 and 11.3 months for DOR and OS, respectively. In total, 61.1% of patients had grade 3 or 4 TRAEs, primarily characterized by a platelet count decline and hypertension. The study exhibited remarkable antitumor activity in aUC patients who were treated with the carelizumab + famitinib combination, particularly among those with UC. This indicated a more favorable treatment response to this combination therapy [117].

Cabozantinib is a multitargeted TKI selectively targeting various receptor kinases implicated in tumor pathogenesis, such as AXL, hepatocyte growth factor receptor (HGFR), and VEGFR. Additionally, this inhibitor affects the tumor immune microenvironment by downregulating regulatory T cells and myeloid suppressor cells. In a phase II study assessing the effectiveness of cabozantinib in 42 mUC patients not responding to platinum treatment, the ORR was 19% (95% CI: 9–34%) [118]. The clinical benefit was observed in 64% of patients (95% CI: 48–79%). Of note, most patients experienced at least 1 dose reduction or dose delay. Moreover, the median OS was 8.1 months (95% CI: 5.2–10.3) during the median follow-up period of 61.2 months [interquartile range (IQR): 53.8–70.0] [118]. The most common grade ≥ 3 AEs were fatigue, hypertension, proteinuria, and hypophosphatemia. Notably, cabozantinib possibly produced favorable results, especially in patients with lung lesions. In a study involving 15 patients with lung metastases, the ORR was 27%, while the SD rate was 73% [118]. Furthermore, an exploratory translational analysis conducted in this study revealed that cabozantinib reduced the number of myeloid suppressor cells, decreased the proportion of regulatory T cells within the overall CD4 T cell population, and increased the percentage of effector CD8 T cells to regulatory T cells. Of note, cabozantinib significantly increased PD-1 expression in regulatory T cells. Based on these findings, cabozantinib combined with ICIs may be a viable therapeutic strategy against aUC [118, 119]. In the clinical trial (ISRCTN25859465), researchers hypothesized that conversion maintenance therapy with cabozantinib improves outcomes in aUC patients who had benefitted from platinum-containing therapy [120]. The patients were randomized to the cabozantinib or placebo group for maintenance treatment. PFS rates were observed in 83.3% and 83.9% of the cabozantinib and placebo groups, respectively. The median PFS for the cabozantinib group was 13.7 weeks (80% CI: 12.1–23.3), while that for the placebo group was 15.8 weeks (80% CI: 11.3–23.6). The adjusted HR for cabozantinib was 0.89 (80% CI: 0.61–1.3, unilateral *P* = 0.35) [118]. Furthermore, no significant difference in OS was observed between the two groups (HR: 0.80, 95% CI: 0.52–1.23, *P* = 0.25). Overall, although cabozantinib was well-tolerated, compared with placebo, it demonstrated no clear benefit when used for maintenance therapy after platinum-based chemotherapy [120].

#### Multitargeting TKI in combination therapies

The multicenter, multicohort, open-label phase Ib/II trial FIDES-02 (NCT04045613) assessed the effectiveness, safety, and tolerability of dovitinib as a monotherapy and combined with atezolizumab [121]. The main aim of this trial was to evaluate the ORR [121].

Another phase II open-label trial was conducted to determine the effectiveness of the axitinib and avelumab combination in 20 aUC patients who had previously failed cisplatin therapy [122]. The trial reported a confirmed ORR of 10.0% in the UC cohort, with all responses being partial. Importantly, the antitumor effect was observed regardless of PD-L1 expression. However, the observed ORR was lower than anticipated, possibly attributable to the study’s limited sample size [122].

In a phase I trial (NCT02496208), the security and effectiveness of cabozantinib and nivolumab (CaboNivo) and CaboNivo combined with ipilimumab (CaboNivoIpi) were evaluated in 54 patients with aUC and other GU malignancies (Table 4) [123]. The patients were included across eight dose levels, with a median follow-up of 44.6 months. Severe TRAEs were noted in 75% and 87% of patients in the CaboNivo and CaboNivoIpi arms, respectively. RP2D was cabozantinib 40 mg/day plus nivolumab 3 mg/kg for CaboNivo and cabozantinib 40 mg/day, nivolumab 3 mg/kg, and ipilimumab 1 mg/kg for CaboNivoIpi. The ORR was 30.6% (95% CI: 20.0–47.5%) and 38.5% (95% CI: 13.9–68.4%) for all patients and those with aUC, respectively. Importantly, CaboNivo and CaboNivoIpi resulted in manageable toxicities, along with durable responses and promising survival results in patients with aUC and other GU tumors [123]. The ARCADIA trial (NCT03824691) determined the effectiveness of the cabozantinib + durvalumab combination in patients with aUC or non-UC variant histologies (VH) who had undergone chemotherapy [124]. In this trial, patients with aUC or non-UC VH who experienced relapse or progression after at least 1 cycle of platinum-based chemotherapy received 40 mg/day orally cabozantinib and 1,500 mg durvalumab intravenously every 4 weeks until deterioration or toxic reactions beyond acceptable levels occurred. The study’s interim results revealed that among the 58 patients who responded, 12 achieved the CR and 11 achieved the PR, which resulted in an ORR of 39.7% (95% CI: 27.1–53.4). The DCR was 69% (95% CI: 55.5–80.5). In the cohort of non-UC VH patients, the ORR was 45% (95% CI: 23.1–68.5), the median PFS was 7.6 months (95% CI: 4.6–13.6), and the median OS was 11.6 months (95% CI: 6.8–20.3). Overall, 35 of the 63 patients (55.5%) experienced various grades of TRAEs, with only 4 patients (6.3%) reporting grade 3 TRAEs. No serious AEs were reported. Approximately 39% of patients required a dose reduction of cabozantinib. Overall, the combination of cabozantinib and durvalumab exhibited promising preliminary activity and controlled security performance in aUC patients as well as in non-UC VH patients who had received chemotherapy [124]. The COSMIC-021 study (NCT03170960) assessed the effectiveness of combination therapy with cabozantinib and atezolizumab in various solid tumors [125]. The study included enrollees from cohorts C3, C4, and C5 with different treatment histories. In cohort C3, which comprised 30 treatment-naïve patients non-eligible for cisplatin therapy, and cohort C4, consisting of 30 patients eligible for cisplatin therapy, a significant clinical benefit was noted with ORRs of 20% and 30%, respectively. In cohort C5 too, which comprised 31 patients who had received 1 prior ICI but no VEGFR-TKI treatment, a benefit from the cabozantinib + atezolizumab combination was noted. Diarrhea, nausea, fatigue, and decreased appetite were the most common TRAEs of any grade in the three cohorts. Severe AEs were reported by 63%, 43%, and 45% of the patients in the respective cohorts. Of note, no grade 5 TRAEs leading to death were reported. The study thus concluded that the combination of cabozantinib and atezolizumab exhibited promising effectiveness and manageable toxicity in inoperable aUC patients. Regardless of the patient’s eligibility for cisplatin-based chemotherapy, this combination therapy holds potential as a first-line systemic therapy for aUC patients. Furthermore, for patients previously treated with ICIs, this combination therapy may also serve as a viable second- or third-line therapy [125]. The ongoing phase II ABATE study (HCRN GU18-343, NCT04289779) investigates the potential of the cabozantinib + atezolizumab combination as a neoadjuvant therapy for MIBC [126]. The trial (NCT03425201) determined the security and effectiveness of the combination of niraparib and cabozantinib against cancers, specifically focusing on aUC [127]. Of the 19 patients enrolled in the study, 14 had aUC. Among the evaluable patients, 3 (16%) aUC patients achieved the PR, while 14 patients (74%) had SD. Preliminary data from the phase I of the study indicated that the aforementioned combination therapy is safe and has manageable toxicity [127]. Phase II studies are currently ongoing and actively recruiting patients to investigate the security and effectiveness of this therapy in GU cancers. A randomized, multicenter, international phase III trial in aUC patients mainly determined the security and effectiveness of the combination of cabozantinib and avelumab as maintenance treatment after initial chemotherapy. The trial also investigated whether the addition of cabozantinib offered superior clinical outcomes compared with avelumab alone [128].

**Table 4:** Ongoing clinical trials of multi-targeting TKIs therapy in UC

**Drugs**	**Targets**	**Combination**	**Conditions**	**Phase**	**NCT**
Anlotinib	VEGFR, FGFR, PDGFR, c-kit	Platinum/Gemcitabine	UC	II	NCT05030077
Axitinib	VEGFR2, PDGFRβ, c-kit,	PF-07265807	Adv/Met solid tumors	I	NCT04458259
Cabozantinib	AXL, HGFR, VEGFR	Durvalumab	UC	II	NCT03824691
Cabozantinib	AXL, HGFR, VEGFR	Atezolizumab	UC	II	NCT04289779
Cabozantinib	AXL, HGFR, VEGFR	Pembrolizumab	Met UC	II	NCT03534804
Cabozantinib	AXL, HGFR, VEGFR	Atezolizumab	UC	I/II	NCT03170960
Cabozantinib	AXL, HGFR, VEGFR	Ipilimumab/Nivolumab	UC	II	NCT03866382
Cabozantinib	AXL, HGFR, VEGFR	Avelumab	Adv /Met UC	III	NCT05092958
Cabozantinib	AXL, HGFR, VEGFR	Nivolumab/Nivolumab + Ipilimumab	Met/Adv UC	I	NCT02496208
Cabozantinib	AXL, HGFR, VEGFR	Enfortumab Vedotin	Met/Adv UC	I	NCT04878029
Cabozantinib	AXL, HGFR, VEGFR	Nivolumab	Bladder Melanoma	II	NCT05111574
Cabozantinib	AXL, HGFR, VEGFR	Nivolumab	Rec UC	I	NCT04514484
Famitinib	c-kit, VEGFR2, PDGFRβ	Camrelizumab	UC	II	NCT03827837

VEGFR: vascular endothelial growth factor receptor; FGFR: fibroblast growth factor receptor; PDGFR: platelet-derived growth factor receptor; HGFR: hepatocyte growth factor receptor; TKIs: tyrosine kinase inhibitors; UC: urothelial carcinoma; Adv: advanced; Met: metastatic; Rec: recurrent; NCT: national clinical trial

## Targeted therapies aiming the PI3K/Akt/mTOR pathway

The mTOR pathway is considered among the most evidently activated signaling pathways in UC [129]. PI3K activation occurs by the binding of various growth factors to their respective receptors, such as those belonging to FGFR and ErbB families. Upon activation, PI3K facilitates Akt1 activation through phosphatidylinositol 3,4,5-trisphosphate (PIP3) [129]. Activated Akt1 inhibited the tuberous sclerosis complex (TSC) [53]. Subsequently, because of the inhibitory function of TSC, Ras homolog protein enriched in brain (Rheb) is mobilized. Once Rheb is no longer inhibited, it activates mTOR. Activated mTOR facilitates cell growth by interacting with various effectors. Phosphatase and tensin homolog (PTEN) is another substantial regulator of the PI3K/Akt/mTOR pathway [129] that functions by inhibiting Akt1 activation through PIP3 dephosphorylation [53].

Mutations in the PI3K/Akt/mTOR pathway are commonly observed in UC. A comprehensive analysis conducted by the TCGA program revealed that 32 genes were significantly altered. Alterations in PI3K/Akt/mTOR pathway-associated genes occurred in 42% of these samples, while alternations in RTK/MAPK pathway-associated genes were found in 44% of the samples [8]. Although PTEN mutations occur in only 3–4% of MIBC cases, PTEN protein expression is prevalently lacking in most patients [8, 130].

Considering the highly altered PI3K/Akt/mTOR signaling pathway and its marked effect on tumor development, along with the comprehensive analysis of existing data, a compelling rationale exists for targeting this signaling cascade with broad therapeutic implications [131]. However, initial studies focusing on targeting the PI3K/Akt/mTOR pathway in UC have generated unsatisfactory outcomes, because only a small patient subset exhibited a response. This could be partly because of the substantial crosstalk between the PI3K/Akt/mTOR and RAS-ERK pathways. Attributable to inter-pathway regulation, PI3K/Akt/mTOR pathway inhibition results in enhanced MAPK pathway activity, thereby promoting metastasis [132–135]. To overcome these barriers, therapeutic methods targeting factors that function through PI3K/Akr/mTOR-dependent mechanisms as well as synergistic with RAS signaling and FGFR3 signaling pathways are promising for a more comprehensive array of therapeutic options.

### PI3K inhibitors

Buparlisib (BKM120), a highly specific and potent pan-class PI3K inhibitor, was evaluated to determine the viability and proof of concept in patients with advanced tumors in a phase I study [136]. Subsequently, a phase II study determined the effectiveness of buparlisib in patients with a platinum refractory aUC [137]. In the initial cohort of 16 patients, genetic alterations in the PI3K/Akt/mTOR pathway were not considered while selecting patients. The reported PFS at 2 months was 54%. Afterward, a cohort was screened for four patients with mutations in phosphatidylinositol-4,5-bisphosphate 3-kinase catalytic subunit alpha (PIK3CA), Akt1, or TSC1. Regrettably, the study was terminated early by the sponsors because of under-recruitment. Of note, none of these patients demonstrated progression at the 8-week assessment, and 38% of the patients encountered substantial AEs necessitating dose reductions, which ultimately resulted in the premature withdrawal of 2 patients from the study.

In preclinical studies, the pan-class I PI3K inhibitor copanlisib could increase the effectiveness of ICIs in syngeneic mouse models of UC, irrespective of whether activation alterations are present in the PI3K pathway [138]. A current phase II study is assessing the combination of copanlisib and avelumab as maintenance therapy for mUC patients after chemotherapy. In this trail, the primary objective is to measure PFS [139].

MPT0L145, a novel PI3K catalytic subunit type 3 (PIK3C3)/FGFR inhibitor, exerts significant antitumor effects against UC cells, which makes it a promising first-in-class candidate for UC treatment [140, 141]. Alpelisib, which has a manageable safety profile and promising initial efficacy, may be useful as a therapeutic option for solid tumors with PIK3CA alterations. This rationalizes combining selective PI3Kα inhibition with other drugs for effectively controlling PIK3CA-mutant tumors [142]. Marqués et al. [143] revealed that the nintedanib + alpelisib combination exerts synergistic antitumor activity. Simultaneous administration of nintedanib and PI3K inhibitors not only overcame UC resistance to nintedanib but also increased its antiangiogenic effects [143].

According to preclinical modeling data, pictilisib significantly inhibits the growth of PIK3CA mutation-harboring UC cells [144, 145]. However, no clinical evidence is currently available for evaluating its effectiveness in this context further.

**Figure 3:**
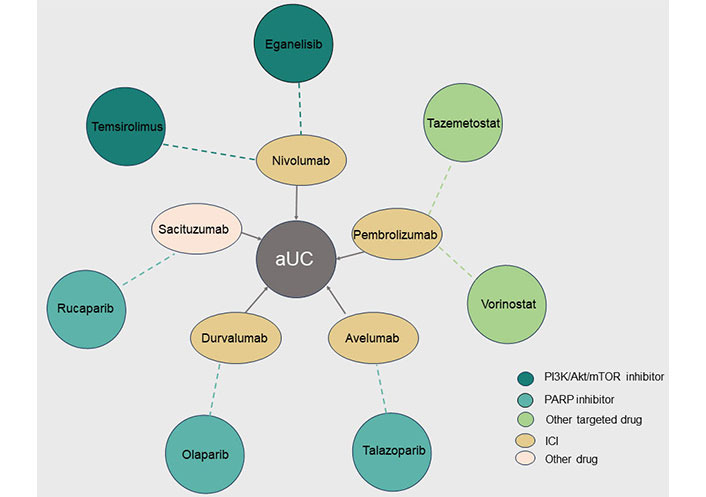
Combination therapies with targeted drugs in advanced urothelial carcinoma (aUC). The different colored circles on the outside represent targeted drugs against different targets, and the ellipses on the inside represent another drug in the combination, with the dotted line indicating the relationship between the combination of two drugs for aUC. PI3K: phosphatidylinositol 3-kinas; Akt: protein kinase B; mTOR: mammalian target of rapamycin; PARP: poly ADP-ribose polymerase; ICI: immune checkpoint inhibitor

Eganelisib (IPI-549) is a novel PI3Kγ inhibitor that is administered orally. In preclinical studies, it exhibited antitumor activity as both monotherapy and in combination with ICIs [146]. The MARIO-1 trial aimed to determine the efficacy of eganelisib as a once-daily single agent and in combination with nivolumab in patients with solid tumors. The trial further determined the security and tolerability of eganelisib compared with that of nivolumab (Figure 3). In the monotherapy arm, the predominant grade ≥ 3 treatment-related toxicities included elevated ALT levels (18%), aspartate aminotransferase (18%), and alkaline phosphatase (5%). During the first 28 days, dose-limiting toxicities (DLTs) were not reported. However, subsequent treatment cycles using 60 mg eganelisib resulted in toxic reactions according to the criteria for DLTs, with the primary occurrence being reversible grade 3 elevations in liver enzymes. In the combination therapy arm, the major grade ≥ 3 treatment-related toxicities included elevated levels of glutamine aminotransferase (13%), ALT, and rash (10%). Overall, 5% of patients receiving monotherapy had serious TRAEs, including 1 case each of grade 4 bilirubin elevation and liver enzyme elevation. Serious TRAEs were observed in 13% of the patients receiving combination therapy. The combination therapy exerted a significant antitumor effect, even in patients whose disease had progressed with the ICIs. Nonetheless, acknowledging that these findings stem from the MARIO-1 trial is crucial, and additional clinical assessments are imperative to validate these results [147].

### Akt inhibitors

Using human UC cell lines, preclinical studies have reported that ectopic pan Akt inhibitors, such as MK-2206 and AZ7328, are potent and selective Akt inhibitors with very low toxicity [148, 149]. Moreover, in preclinical studies, Akt and ERK1/2 phosphorylation in UC cells was significantly increased by the chemotherapeutic agent piroxicam. MK-2206 used as a single agent or in combination with the ERK1/2 inhibitor AZD6244 exhibited significant sensitization of UC cells to the chemotherapeutic agent pirarubicin [150]. Additionally, capivasertib (AZD5363) exhibited UC inhibition in preclinical studies [151]. The ATP-competitive pan-Akt inhibitor ipatasertib (GDC-0068) was evaluated in a phase Ib clinical trial involving aUC patients [152]. The trial revealed that ipatasertib, when combined with chemotherapy or hormonal therapy, exhibits a good tolerability and safety profile consistent with those of other ATP-competitive Akt inhibitors [152].

### mTOR inhibitors

Rapamycin represents the initial discovery of a mTOR inhibitor [153]. This compound specifically targets the mTOR complex 1 (mTORC1), a key regulatory factor in translation and cell growth [153]. In addition to rapamycin, the FDA has approved three other rapamycin analogs, namely everolimus, temsirolimus, and ridaforolimus, which are commonly used. Although, in preclinical studies, everolimus demonstrated antitumor activity, clinical trials have usually reported unsatisfactory results in aUC patients [154]. In a phase II trial, all 45 mUC patients, who had previously received at least one cytotoxic drug and experienced disease progression, received everolimus [155]. The PFS rate at the 2-month mark was 51%. Two patients, however, attained PR, with 1 patient achieving CR for a duration of > 2 years. A post-analysis of the tumor genome in this patient revealed the presence of TSC1 inactivating mutations alongside neurofibromatosis type 2 mutations [156].

In a phase II trial, everolimus was assessed in aUC patients who had experienced progression after chemotherapy [157]. The 2-month DCR in this trial was 27%. PTEN was expressed in all patients with controlled disease (*n* = 6) and 6 of the 14 patients with uncontrolled disease (43%). In a subsequent analysis of archival tissue, Akt activation in PTEN-deficient cells increased during treatment with mTOR inhibitors [157]. Tumors lacking PTEN exhibited heightened Akt activation after treatment with mTOR inhibitors, potentially serving as a mechanism of resistance to everolimus. In addition to PTEN deficiency, increased Akt activity may occur because everolimus selectively inhibits a mTORC1 subunit, leading to increased activation of mTORC2, a known Akt activator [158]. Another clinical study of everolimus in UC reported unfavorable outcomes [159]. However, some case reports have indicated the significant effectiveness of everolimus treatment in patients with PIK3CA mutant UC. This suggests that the rare M1043I mutation variant could potentially serve as a biomarker for everolimus sensitivity [160]. Xia et al. [161] revealed that high-dose everolimus monotherapy causes tumor regression but also induces immunosuppression. The combination of low-dose everolimus with ICIs effectively suppressed UC growth by boosting the antitumor immune response in the tumor microenvironment and peripheral area [161].

In preclinical models, temsirolimus improved the cytotoxic effectiveness of cisplatin and gemcitabine against UC cell lines [162]. A phase II trial used temsirolimus in aUC patients undergoing first-line chemotherapy [163]. Of the 45 patients included, 48.9% patients demonstrated a progression-free status at the 2-month mark. Importantly, > 50% of the patients experienced severe toxicity, and 11 patients had to be discontinued because of the development of adverse effects, which resulted in the discontinuation of recruitment. In addition, another trial investigating temsirolimus as a treatment for aUC patients was prematurely terminated because of insufficient observed efficacy [164].

Second-generation mTOR inhibitors include small-molecule ATP analogs and dual PI3K/mTOR inhibitors. By directly binding to the ATP-binding site of mTOR or PI3K, or by acting upon it, these inhibitors cause competitive inhibition [165]. Three UC cell lines were treated with OSI-027 to effectively hinder the phosphorylation of components within both mTORC1 and mTORC2 pathways, thereby causing a noticeable reduction in cell proliferation. Furthermore, in an *in vitro* assay of UC, OSI-027 combined with lapatinib exerted synergistic antitumor effects. The study also exhibited antitumor synergy in UC *in vitro* [166].

Sapanisertib (TAK-228) inhibits mTORC1 and mTORC2 complexes, and when combined with paclitaxel or the PI3Kα inhibitor TAK-117, sapanisertib exerts synergistic antitumor effects in preclinical UC models [167]. Sapanisertib demonstrated a manageable safety profile in a trial [168]. A phase II clinical trial assessing the efficacy of sapanisertib in aUC patients carrying TSC1 or TSC2 mutations was prematurely terminated because of a lack of effectiveness and a high incidence of AEs [169].

Dactolisib, an oral dual mTOR/PI3K inhibitor, increases the antitumor effect of cisplatin by exerting synergistic effects [170]. A phase II study determined the effectiveness of dactolisib in UC [171]. The study revealed a modest clinical effect, with 10% of patients achieving PFS after 16 weeks. However, the trial also reported a significant level of toxicity.

### PI3K/Akt/mTOR pathway inhibitors in combination therapies

The observed upregulation of RTKs along with the inhibition of the PI3K/Akt/mTOR pathway suggests that inhibitors targeting both pathways may together increase efficacy. Everolimus and pazopanib, a VEGFR inhibitor, were assessed in a phase I trial of aUC patients. Only 21% of the participants had a response. *TSC1/2* or *mTOR* gene alterations were observed among patients who experienced clinical effects. Specifically, of the 5 patients who benefited from the treatment, 4 patients had the aforementioned mutations, whereas the other patient had an FGFR3-TACC3 fusion [112]. A phase Ib study assessed the combined inhibition of PIK3CA and FGFR in patients with different PIK3CA-mutant solid tumors [172]. In that study, 60% of patients encountered severe AEs. Notably, only 10% of patients demonstrated DLTs. Akt and mTOR inhibitors together exert an inhibitory effect on UC cell lines [151]. Specifically, the J82 cell line, which has PI3KCA and mTOR mutations, was sensitive to AZD5363, AZD2014, and BEZ235 individually, as well as the combinations of AZD5363/AZD2014 and AZD5363/BEZ235. Although all single agents exert inhibitory effects on cell proliferation, the combinations exert a synergistic effect on cell viability and colony formation.

## Targeted therapies aiming MAPK pathway inhibitors

The MAPK signaling pathway is critical in cell proliferation, growth, and survival. RTK activates the intracellular RAS GTPase, which then activates rapidly accelerated fibrosarcoma (RAF) to activate the MAPK downstream pathway [173]. Approximately 2–5% of UC patients have *RAS* alterations, whereas V-RAF murine sarcoma viral oncogene homolog B1 (*BRAF*) mutations are present in 2% of patients [174]. Although the MAPK pathway undergoes genetic alteration at a lower frequency in UC than other potential therapeutic targets, its clinical significance lies in its interactions with commonly altered pathways and receptors.

Tipifarnib, a farnesyltransferase inhibitor, effectively reduces the RAS function. However, in a phase II clinical study, the ORR of tipifarnib as a monotherapy for UC was nonsignificant, and no further studies were required [175]. In a phase II clinical trial, tipifarnib was assessed in mUC patients harboring harvey RAS viral oncogene homolog (*HRAS*) mutations [176]. Of the 21 patients, only 19% exhibited PFS at 6 months, which indicated limited efficacy. Sorafenib is a kinase inhibitor targeting various intracellular signaling proteins, particularly RAF. It exerts antiproliferative, antiangiogenic, and proapoptotic properties in tumor cells [177]. According to preclinical studies using UC cell lines, low sorafenib concentrations promote migration and proliferation, whereas high concentrations induce pro-apoptotic effects [177]. A phase II trial evaluated the effectiveness of sorafenib as a monotherapy for aUC patients in the second-line setting. This trial’s results indicated minimal sorafenib activity in this patient population [178]. Another phase I trial investigated the combination of sorafenib and vincristine in post-platinum mUC patients, demonstrating an overall remission rate of 41% [179]. A phase II trial evaluated the effectiveness of the combination of sorafenib, gemcitabine, and carboplatin as a first-line treatment for aUC [180]. Despite reporting a median PFS of 9.5 months, the treatment regimen induced significant toxicity, which led to treatment discontinuation in 65% of patients from the cohort [180]. Therefore, phase III studies are required to further evaluate the combination of sorafenib and vincristine or gemcitabine/carboplatin. Additional clinical trials of sorafenib for UC treatment are ongoing (Table 5).

**Table 5:** Ongoing clinical trials of PI3K/Akt/mTOR and MAPK pathway-targeted therapy in UC

**Drugs**	**Taegets**	**Combination**	**Conditions**	**Phase**	**NCT**
Copanlisib	PI3Kα/δ	Avelumab	Adv UC	I/II	NCT05687721
Ipatasertib	Akt	Null	Adv UC	II	NCT02465060
Sapanisertib	mTOR1/2	Null	Adv UC	II	NCT03047213
Tipifarnib	RAS	Null	UC	II	NCT02535650
Sorafenib	RAF	gemcitabine and cisplatin	UC	II	NCT01222676
Vistusertib	mTOR	Null	UC | MIBC	I	NCT02546661

Null in combination indicates that the trial is monotherapy. MAPK: mitogen-activated protein kinase; UC: urothelial carcinoma; PI3K: phosphatidylinositol 3-kinas; Akt: protein kinase B; mTOR: mammalian target of rapamycin; MIBC: muscle-invasive bladder cancer; Adv: advanced; RAS: sat sarcoma; RAF: rapidly accelerated fibrosarcoma; NCT: National Clinical Trial; |: and

## Targeted therapies aiming poly ADP-ribose polymerase inhibitors

Alterations in DDR genes are also common in UC. Excision Repair Cross-Complementation Group 1 and Excision Repair Cross-Complementation Group 2 are components of the nucleotide excision repair pathway associated with UC [8]. Poly ADP-ribose polymerase (PARP) inhibitors are targeted therapies for UC with *DDR* gene mutations. This targeted therapy was developed based on tumor cell lethality induced by defective DNA repair mechanisms. Preclinical studies have investigated the effects of PARP inhibitors (olaparib, niraparib, rucaparib, veliparib, or talazoparib) alone and their combination with cisplatin on UC cells [181]. Niraparib and talazoparib as single agents effectively reduced the UC cell survival. Moreover, cisplatin combined with talazoparib and niraparib significantly reduced UC cell survival, whereas veliparib exhibited limited effectiveness even at high concentrations. In *in vivo* experiments, cisplatin combined with niraparib, rucaparib, or talazoparib significantly reduced the growth of UC xenografts. The accumulated evidence suggests that PARP inhibitors exhibit efficacy for UC as single agents or in combination with cisplatin [181].

The trial ATLAS demonstrated that rucaparib exhibited no significant effect in previously treated aUC patients, irrespective of the tumor’s homologous recombination defect status [182]. However, a recent phase II study used rucaparib to evaluate US patients who were positive for DNA repair deficiency biomarkers after chemotherapy [183]. The findings indicated that rucaparib continued after platinum-based chemotherapy prolonged survival in aUC patients screened for the levels of DNA repair deficiency biomarkers, and the treatment was well tolerated. Therefore, further studies investigating the use of PARP inhibitors in selected aUC patients are warranted. Clinical studies have exhibited promising antitumor activity when rucaparib was combined with sacituzumab and govitecan in patients with advanced solid tumors. Regardless of the presence of homologous recombination repair (*HRR*) gene alterations, these findings include the results of tumor patients treated with PARP inhibitors. A phase II study demonstrated limited antitumor activity when olaparib was given as a monotherapy in patients with altered aUC and DDR [184].

A neoadjuvant therapy trial demonstrated the tolerability and effectiveness of the Olaparib + durvalumab combination, with a pathologic CR rate of 50% at cystectomy [185]. The trial BAYOU investigated the impact of the durvalumab + olaparib combination in patients with untreated mUC non-eligible for platinum therapy [186]. However, concomitant use of olaparib and durvalumab caused no improvement in patient survival in an unselected population of aUC patients. The efficacy outcomes observed with durvalumab were comparable to those reported for other PD-1/PD-L1-targeting agents. However, secondary analyses of the results indicated a potential role for PARP inhibition in UC patients with *HRR* gene mutations. The Meet-URO12 trial assessed the addition of niraparib as maintenance therapy to optimal supportive treatment for aUC patients with no disease progression after chemotherapy [187]. No significant difference was observed in terms of PFS between the niraparib and placebo groups. This study did not specify the presence of *DDR* gene alterations for enrollment. Although no increase in PFS was observed in this patient group with *HRR* gene alterations, a larger sample size may be required to observe any potential alterations [187].

The non-randomized controlled JAVELIN PARP Medley trial determined the therapeutic effectiveness of the avelumab and tazopanib combination in patients with advanced solid tumors [188]. The ORR of the combination therapy (avelumab + tazopanib) was comparable to that of monotherapy with PARP inhibitors or ICI [188]. Currently, a phase II trial (NCT04678362) is evaluating talazoparib and avelumab to determine their effectiveness and security for use as maintenance therapies in aUC patients sensitive to platinum therapy [189]. Currently, the use of PARP inhibitor therapy has not yielded promising results in UC management. However, optimizing patient selection can potentially increase treatment outcomes. Additional studies are warranted to identify patients who are most likely to benefit from PARP inhibitors. Moreover, ongoing investigations examining the efficacy of PARP inhibitors in novel clinical settings hold promise for improved treatment outcomes (Table 6).

**Table 6:** Ongoing clinical trials of PARP-targeted therapy in UC

**Drugs**	**Targets**	**Combinations**	**Conditions**	**Phase**	**NCT**
Niraparib	PARP	Dostarlimab	UC	II	NCT04779151
Niraparib	PARP	Atezolizumab	UC	I/II	NCT03869190
Olaparib	PARP	Null	Met/Adv UC	II	NCT03375307
Olaparib	PARP	EP0057	UC	I/II	NCT02769962
Olaparib	PARP	Durvalumab	UC | MIBC	I	NCT02546661
Olaparib	PARP	Ceralasertib	Met UC	II	NCT03682289
Talazoparib	PARP	Avelumab	UC	II	NCT04678362
Veliparib	PARP	Paclitaxel, carboplatin	UC	I	NCT01366144

Null in combination indicates that the trial is monotherapy. PARP: poly ADP-ribose polymerase; MIBC: muscle-invasive bladder cancer; UC: urothelial carcinoma; Adv: advanced; Met: metastatic. |: and

## Other targeted therapies

### Targeted therapies aiming chromatin remodeling

Before ICIs were introduced, the standard second-line therapy remained undefined for aUC patients. HDAC inhibitors have demonstrated anticancer effects in various tumor models, including the modulation of apoptosis of UC cell lines. Vorinostat is an enzyme inhibitor that significantly impedes cell survival, growth, and apoptosis regulation, all of which are essential in cancer. Consequently, this drug can disrupt a tumor’s capability to sustain its growth. A phase II study determined the response rate to vorinostat in aUC patients. The patients received 200 mg vorinostat [suberoylanilide hydroxamic acid (SAHA)] orally each day on a 3-week cycle. All 14 patients completed the study. The OS was 4.3 months (95% CI: 2.1–8.3), while the PFS was 1.1 months (95% CI: 1.0–2.1). The study was terminated early after the first stage of a two-stage design, which allowed for discontinuation because of unsatisfactory results (NCT00363883). A phase I study investigated the combination of vorinostat and docetaxel in patients with advanced and relapsed solid malignancies, including UC. The trial determined whether this combination treatment yielded outcomes superior to those of docetaxel alone (NCT00565227). However, the study was unfortunately terminated prematurely, and no results have been published. The trial (NCT00363883) assessed the effectiveness and toxicity of vorinostat in aUC patients who experienced treatment failure with first-line therapy, in the adjuvant/neoadjuvant setting, or for recurrent/advanced tumors [190]. The main endpoint was the response rate. A response rate of > 20% was considered notable for the two-stage study design, which required at least one response in the initial 12 patients so as to proceed to the second stage, with a total of 37 subjects. In the absence of any observed response, the initial phase of recruitment was terminated. Among the patients, the best response of SD was achieved in three individuals. Because of the limited efficacy and the notable toxicity associated with vorinostat at this specific dosage regimen, the risk-benefit ratio was unsatisfactory for aUC patients.

A phase I/Ib study determined the effectiveness of the combination of pembrolizumab and vorinostat for aUC patients, but the study results have not been presented at this time (NCT02619253).

Through preclinical experiments, Michael et al. [191] first demonstrated the ability of the HDAC inhibitor belinostat to effectively suppress UC cell growth. Alterations in COMPASS-associated proteins were noted in approximately two-thirds of UC patients, which suggested that histone regulation is associated with tumor survival [192]. Administering the enhancer of zeste homolog 2 inhibitor tazemetostat to mice reduced UC disease. Moreover, tazemetostat administered *in vivo* boosted the immune response by directly affecting cytokines and antigen recognition mechanisms, particularly major histocompatibility complex I (MHC-I) and MHC-II [192]. Consequently, a pilot study (ETCTN 10183; NCT03854474) was conducted to evaluate the effectiveness of the tazemetostat and pembrolizumab combination against aUC (Table 7) [192]. The study comprised two cohorts: cohort A involving patients refractory to cisplatin, while cohort B included patients non-eligible for chemotherapy. Each cohort consisted of 12 patients. The treatment regimen involved 800 mg tazemetostat administered twice daily in conjunction with 200 mg pembrolizumab every 3 weeks. The median treatment duration was 12 weeks (IQR: 7–30; range: 3–107 weeks). In this study, 1 case of grade 4 sepsis and 2 cases of grade 3 lymphopenia were reported. Other observed TRAEs included 1 case each of anemia, elevated alkaline phosphatase, and herpes simplex virus oral infections. In this study, three patients (25%) achieved PR, while three (25%) experienced SD [192]. The median survival was 3.1 months (95% CI: 2.3–NA), with a median OS of 8.0 months (95% CI: 4.7–NE). The study successfully determined that RP2D is the combination of 800 mg tazemetostat and 200 mg pembrolizumab administered every 3 weeks. This therapy demonstrated durable effects and was feasible and well tolerated in patients with low-risk chemotherapy-refractory UC.

**Table 7:** Ongoing clinical trials of other targeted therapies in UC

**Drugs**	**Targets**	**Comnonation**	**Conditions**	**Phase**	**NCT**
Abemaciclib	CDK4/6	Null	UC	I	NCT03837821
Belinostat	HDAC	Durvalumab + tremelimumab	UC	I	NCT05154994
Tazemetostat	EZH2	Pembrolizumab	UC	I/II	NCT03854474
Trilaciclib	CDK4/6	Gemcitabine + cisplatin/carboplatin + avelumab	UC	II	NCT04887831
Vactosertib	TGF-β	Durvalumab	UC	II	NCT04064190
Vorinostat	HDAC 1, 2, 3, 6, 7, 11	Docetaxel	UC	I	NCT00565227

Null in Combination indicates that the trial is monotherapy. UC: urothelial carcinoma; CDK4/6: cyclin-dependent kinases 4 and 6; HDAC: histone deacetylase; EZH2: enhancer of zeste homolog; TGF-β: transforming growth factor β

### Targeted therapies aiming cell cycle regulation

Cell cycle regulation is another crucial target of UC. The cyclin-dependent kinase inhibitor 2A/B (*CDKN2A/B*) gene, responsible for inhibiting p14 and p16 of the cyclin-dependent kinases 4 and 6 (CDK4/6), is commonly altered in 5–23% of MIBC cases [8, 56]. CDK4/6 inhibition has emerged as a therapeutic target in UC. Palbociclib is a highly selective, orally administered CDK4 and CDK6 inhibitor. Inhibition of these proteins facilitates the restoration of the tumor suppressor effects of retinoblastoma protein (RB), thereby leading to cell cycle arrest. The crucial role of an intact RB in the mechanism underlying CDK4/6 inhibitor therapy in cancer is well-established. The presence of CDKN2A deletion with an intact RB mechanistically predicts sensitivity to CDK4/6 inhibitors. According to preclinical data from UC cell lines, RB1 inactivation leads to resistance, while CDKN2A inactivation results in sensitivity to palbociclib [193]. An open-label, single-arm, multicenter phase II trial was conducted to determine the effectiveness of palbociclib in previously treated UC patients exhibiting p16 deletion and an intact RB. Regrettably, palbociclib exhibited ineffectiveness, as only 17% of patients achieved PFS at 4 months [194]. Preclinical studies have reported that the combination of palbociclib and talazoparib enhances treatment efficacy in UC, although additional clinical studies are warranted to validate these findings [195]. Trilaciclib and abemaciclib have also entered clinical trials and are being assessed for their effects on UC.

### Emerging therapeutic targets

Evidence suggests that the transforming growth factor β (TGF-β) is a critical factor for cancer that influences various aspects, including resistance to cell death, evasion of growth inhibitors, induction of angiogenesis, and activation of invasion and metastasis [196]. Furthermore, the TGF-β pathway serves as a modulator of immune cell rejection and a contributor to ICI resistance [196]. The effectiveness of TGF-β pathway inhibitors in combination with ICIs against various tumor types, including UC, has been assessed. Unfortunately, two clinical trials investigating the combination of SAR439459 and cemiplimab for treating patients with advanced solid tumors were prematurely terminated because of drug toxicity. An ongoing phase II study (NCT04064190) aims to assess the potential of the vactosertib + durvalumab combination in significantly improving the ORR among UC patients who have not achieved remission with an anti-PD-1/PD-L1 based regimen. The aryl hydrocarbon receptor (AHR), a transcription factor, binds to diverse endogenous and exogenous ligands and thus modulates the function of various innate and adaptive immune cells. Elevations in AHR levels have been linked to resistance against ICIs. In preclinical studies, the selective orally active AHR antagonist called IK-175 could impede tumor growth and reverse immunosuppression in mouse tumor models [197]. According to preliminary analyses, both the monotherapy and combination therapy arms of IK-175 demonstrated promising antitumor activity and good tolerability in eligible UC patients [198]. During a phase II trial, aUC patients whose disease had recurred or progressed after chemotherapy received a combination therapy involving the soluble ephrin type-B receptor 4-human serum albumin (sEphB1-HSA) and pembrolizumab. The combined use of sEphB1-HSA and pembrolizumab had synergistic effects, which improved OS and ORR [197]. Additionally, clinical trials evaluating the combination of arginase inhibitor INCB001158 and the burton TKI acalabrutinib with ICIs for UC patients are currently in progress.

## Conclusion

A rapid evolution in the systemic therapy of UC has recently occurred, encompassing various therapeutic approaches such as chemotherapy, immunotherapy, targeted therapy, and antibody conjugate therapy. Because our understanding of key genetic alterations in UC pathogenesis has advanced rapidly, the potential for breakthroughs has intensified. Targeted therapies for several targets, including TK receptors, intracellular pathways, and intranuclear processes, are currently being developed. Among these therapies, erdafitinib is the most effective targeted therapy and has received FDA approval for use in aUC management. Advancements are being made in targeting RTK inhibitors, and inhibitors for PI3K/Akt/mTOR, MAPK, and PARP pathways. Simultaneously, combining targeted agents with ICIs, other pathway-targeting agents, or different drug types exhibited treatment efficacy against aUC patients, with numerous promising drug combinations currently being investigated in clinical trials. Owing to the ongoing advancements in tumor molecular characterization and the continuous progress of clinical trials, UC patients can expect to apply more targeted therapy combinations for enhancing prognosis.

## Abbreviations

ADC: antibody-drug conjugate
AE: adverse event
AHR: aryl hydrocarbon receptor
Akt: protein kinase B
ALT: alanine aminotransferase
aUC: advanced urothelial carcinoma
BC: bladder cancer
CaboNivo: cabozantinib and nivolumab
CaboNivoIpi: cabozantinib and nivolumab combined with ipilimumab
CD: continuous dose
CDK4: cyclin-dependent kinases 4
CDKN2A: cyclin-dependent kinase inhibitor 2A
CET: cetrelimab
CI: confidence interval
CR: complete response
DCR: disease control rate
DDR: DNA damage response
DLTs: dose-limiting toxicities
DOR: duration of remission
EGFR: epidermal growth factor receptor
ErbB: erythroblastic oncogene B
ERK: extracellular signal-regulated kinase
FDA: Food and Drug Administration
FGF: fibroblast growth factors
FGFR: fibroblast growth factor receptor
GU: genitourinary
HDAC: histone deacetylase
HER1: human epidermal growth factor receptor 1
HR: hazard ratio
HRR: homologous recombination repair
ICIs: immune checkpoint inhibitors
ID: intermittent dose
IHC: immunohistochemistry
MAPK: mitogen-activated protein kinase
MIBC: muscle-invasive bladder cancer
mTOR: mammalian target of rapamycin
mTORC1: mechanistic target of rapamycin complex 1
mUC: metastatic urothelial carcinoma
NE: not estimable
ORR: objective response rate
OS: overall survival
P4HA2: prolyl 4-hydroxylase subunit α2
PARP: poly ADP-ribose polymerase
PD-1: programmed cell death protein 1
PDGFR: platelet-derived growth factor receptor
PD-L1: programmed cell death ligand 1
PFS: progression-free survival
PI3K: phosphatidylinositol 3-kinase
PR: partial response
PTEN: phosphatase and tensin homolog
RAF: rapidly accelerated fibrosarcoma
RAS: rat sarcoma
RB: retinoblastoma protein
RC48-ADC: disitamab vedotin
RP2D: recommended phase II dose
RTKs: receptor tyrosine kinases
SD: stable disease
TACC3: transforming acidic coiled-coil containing protein 3
T-DM1: trastuzumab-emtansine
T-DXd: trastuzumab deruxtecan
TGF-β: transforming growth factor β
TK: tyrosine kinase
TKI: tyrosine kinase inhibitor
TRAE: treatment-related adverse event
TSC: tuberous sclerosis complex
UC: urothelial carcinoma
VEGF-A: vascular endothelial growth factor-A
VEGFR: vascular endothelial growth factor receptor
VH: variant histologies

## Declarations

### Copyright

© The Author(s) 2024.

## References

[1] Sung H, Ferlay J, Siegel RL, Laversanne M, Soerjomataram I, Jemal A (2021). Global cancer statistics 2020: GLOBOCAN estimates of incidence and mortality worldwide for 36 cancers in 185 countries. CA Cancer J Clin.

[2] Babjuk M, Burger M, Capoun O, Cohen D, Compérat EM, Dominguez Escrig JL (2022). European association of urology guidelines on non-muscle-invasive bladder cancer (Ta, T1, and Carcinoma in Situ). Eur Urol.

[3] Burger M, Catto JW, Dalbagni G, Grossman HB, Herr H, Karakiewicz P (2013). Epidemiology and risk factors of urothelial bladder cancer. Eur Urol.

[4] Siegel RL, Miller KD, Fuchs HE, Jemal A (2022). Cancer statistics, 2022. CA Cancer J Clin.

[5] Galsky MD, Hahn NM, Rosenberg J, Sonpavde G, Hutson T, Oh WK (2011). Treatment of patients with metastatic urothelial cancer “unfit” for cisplatin-based chemotherapy. J Clin Oncol.

[6] Maisch P, Hwang EC, Kim K, Narayan VM, Bakker C, Kunath F (2023). Immunotherapy for advanced or metastatic urothelial carcinoma. Cochrane Database Syst Rev.

[7] Robertson AG, Kim J, Al-Ahmadie H, Bellmunt J, Guo G, Cherniack AD (2017). Comprehensive molecular characterization of muscle-invasive bladder cancer. Cell.

[8] (2014). Cancer Genome Atlas Research Network. Comprehensive molecular characterization of urothelial bladder carcinoma. Nature.

[9] Kamoun A, de Reyniès A, Allory Y, Sjödahl G, Robertson AG, Seiler R (2020). A consensus molecular classification of muscle-invasive bladder cancer. Eur Urol.

[10] Zangouei AS, Barjasteh AH, Rahimi HR, Mojarrad M, Moghbeli M (2020). Role of tyrosine kinases in bladder cancer progression: an overview. Cell Commun Signal.

[11] Moghbeli M, Makhdoumi Y, Soltani Delgosha M, Aarabi A, Dadkhah E, Memar B (2019). ErbB1 and ErbB3 co-over expression as a prognostic factor in gastric cancer. Biol Res.

[12] Goetz R, Mohammadi M (2013). Exploring mechanisms of FGF signalling through the lens of structural biology. Nat Rev Mol Cell Biol.

[13] Klint P, Claesson-Welsh L (1999). Signal transduction by fibroblast growth factor receptors. Front Biosci.

[14] Helsten T, Schwaederle M, Kurzrock R (2015). Fibroblast growth factor receptor signaling in hereditary and neoplastic disease: biologic and clinical implications. Cancer Metastasis Rev.

[15] Katoh M (2016). FGFR inhibitors: effects on cancer cells, tumor microenvironment and whole-body homeostasis (review). Int J Mol Med.

[16] Nassar AH, Lundgren K, Pomerantz M, Van Allen E, Harshman L, Choudhury AD (2018). Enrichment of FGFR3-TACC3 fusions in patients with bladder cancer who are young, asian, or have never smoked. JCO Precis Oncol.

[17] Hood FE, Royle SJ (2011). Pulling it together: the mitotic function of TACC3. Bioarchitecture.

[18] Nelson KN, Meyer AN, Siari A, Campos AR, Motamedchaboki K, Donoghue DJ (2016). Oncogenic gene fusion FGFR3-TACC3 is regulated by tyrosine phosphorylation. Mol Cancer Res.

[19] Parker BC, Engels M, Annala M, Zhang W (2014). Emergence of FGFR family gene fusions as therapeutic targets in a wide spectrum of solid tumours. J Pathol.

[20] Parker BC, Annala MJ, Cogdell DE, Granberg KJ, Sun Y, Ji P (2013). The tumorigenic *FGFR3-TACC3* gene fusion escapes miR-99a regulation in glioblastoma. J Clin Invest.

[21] Costa R, Carneiro BA, Taxter T, Tavora FA, Kalyan A, Pai SA (2016). *FGFR3-TACC3* fusion in solid tumors: mini review. Oncotarget.

[22] Tomlinson DC, Baldo O, Harnden P, Knowles MA (2007). FGFR3 protein expression and its relationship to mutation status and prognostic variables in bladder cancer. J Pathol.

[23] Billerey C, Chopin D, Aubriot-Lorton MH, Ricol D, Gil Diez de Medina S, Van Rhijn B (2001). Frequent *FGFR3* mutations in papillary non-invasive bladder (pTa) tumors. Am J Pathol.

[24] di Martino E, Tomlinson DC, Knowles MA (2012). A decade of FGF receptor research in bladder cancer: past, present, and future challenges. Adv Urol.

[25] Helsten T, Elkin S, Arthur E, Tomson BN, Carter J, Kurzrock R (2016). The FGFR landscape in cancer: analysis of 4,853 tumors by next-generation sequencing. Clin Cancer Res.

[26] Tomlinson DC, Baxter EW, Loadman PM, Hull MA, Knowles MA (2012). FGFR1-induced epithelial to mesenchymal transition through MAPK/PLCγ/COX-2-mediated mechanisms. PLoS One.

[27] Perera TPS, Jovcheva E, Mevellec L, Vialard J, De Lange D, Verhulst T (2017). Discovery and pharmacological characterization of JNJ-42756493 (Erdafitinib), a functionally selective small-molecule FGFR family inhibitor. Mol Cancer Ther.

[28] Siefker-Radtke AO, Necchi A, Park SH, García-Donas J, Huddart RA, Burgess EF (2022). Efficacy and safety of erdafitinib in patients with locally advanced or metastatic urothelial carcinoma: long-term follow-up of a phase 2 study. Lancet Oncol.

[29] Loriot Y, Necchi A, Park SH, Garcia-Donas J, Huddart R, Burgess E (2019). Erdafitinib in locally advanced or metastatic urothelial carcinoma. N Engl J Med.

[30] Loriot Y, Necchi A, Park SH, Huddart RA, Burgess E, Zhong B (2018). Erdafitinib compared with vinflunine or docetaxel or pembrolizumab in patients (pts) with metastatic or surgically unresectable (M/UR) urothelial carcinoma (UC) and selected fgfr gene alterations (FGFRalt): the phase III THOR study. Ann Oncol.

[31] Loriot Y, Matsubara N, Park SH, Huddart RA, Burgess EF, Houede N (2023). Phase 3 THOR study: results of erdafitinib (erda) versus chemotherapy (chemo) in patients (pts) with advanced or metastatic urothelial cancer (mUC) with select fibroblast growth factor receptor alterations (FGFRalt). J clin Oncol.

[32] Li X, Li Y, Liu B, Chen L, Lyu F, Zhang P (2023). P4HA2-mediated HIF-1α stabilization promotes erdafitinib-resistance in FGFR3-alteration bladder cancer. Faseb J.

[33] Facchinetti F, Hollebecque A, Braye F, Vasseur D, Pradat Y, Bahleda R (2023). Resistance to selective FGFR inhibitors in *FGFR*-driven urothelial cancer. Cancer Discov.

[34] Schuler M, Cho BC, Sayehli CM, Navarro A, Soo RA, Richly H (2019). Rogaratinib in patients with advanced cancers selected by FGFR mRNA expression: a phase 1 dose-escalation and dose-expansion study. Lancet Oncol.

[35] Grünewald S, Politz O, Bender S, Héroult M, Lustig K, Thuss U (2019). Rogaratinib: a potent and selective pan-FGFR inhibitor with broad antitumor activity in FGFR-overexpressing preclinical cancer models. Int J Cancer.

[36] Sternberg CN, Petrylak DP, Bellmunt J, Nishiyama H, Necchi A, Gurney H (2022). FORT-1: phase II/III study of rogaratinib versus chemotherapy in patients with locally advanced or metastatic urothelial carcinoma selected based on *FGFR1/3* mRNA expression. J Clin Oncol.

[37] Liu PCC, Koblish H, Wu L, Bowman K, Diamond S, DiMatteo D (2020). INCB054828 (pemigatinib), a potent and selective inhibitor of fibroblast growth factor receptors 1, 2, and 3, displays activity against genetically defined tumor models. PLoS One.

[38] Subbiah V, Iannotti NO, Gutierrez M, Smith DC, Féliz L, Lihou CF (2022). FIGHT-101, a first-in-human study of potent and selective FGFR 1-3 inhibitor pemigatinib in pan-cancer patients with *FGF/FGFR* alterations and advanced malignancies. Ann Oncol.

[39] Guagnano V, Furet P, Spanka C, Bordas V, Le Douget M, Stamm C (2011). Discovery of 3-(2,6-dichloro-3,5-dimethoxy-phenyl)-1-{6-[4-(4-ethyl-piperazin-1-yl)-phenylamino]-pyrimidin-4-yl}-1-methyl-urea (NVP-BGJ398), a potent and selective inhibitor of the fibroblast growth factor receptor family of receptor tyrosine kinase. J Med Chem.

[40] Nogova L, Sequist LV, Perez Garcia JM, Andre F, Delord JP, Hidalgo M (2017). Evaluation of BGJ398, a fibroblast growth factor receptor 1-3 kinase inhibitor, in patients with advanced solid tumors harboring genetic alterations in fibroblast growth factor receptors: results of a global phase I, dose-escalation and dose-expansion study. J Clin Oncol.

[41] Pal SK, Rosenberg JE, Hoffman-Censits JH, Berger R, Quinn DI, Galsky MD (2018). Efficacy of BGJ398, a fibroblast growth factor receptor 1–3 inhibitor, in patients with previously treated advanced urothelial carcinoma with FGFR3 alterations. Cancer Discov.

[42] Lyou Y, Grivas P, Rosenberg JE, Hoffman-Censits J, Quinn DI, D PP (2020). Hyperphosphatemia secondary to the selective fibroblast growth factor receptor 1–3 inhibitor infigratinib (BGJ398) is associated with antitumor efficacy in fibroblast growth factor receptor 3–altered advanced/metastatic urothelial carcinoma. Eur Urol.

[43] Grivas P, Daneshmand S, Makarov V, Bellmunt J, Sridhar SS, Sonpavde GP (2023). Fibroblast growth factor receptor 3 (*FGFR3*) alterations in PROOF 302: A phase III trial of infigratinib (BGJ398) as adjuvant therapy in patients (pts) with invasive urothelial carcinoma (UC). J Clin Oncol.

[44] Sootome H, Fujita H, Ito K, Ochiiwa H, Fujioka Y, Ito K (2020). Futibatinib is a novel irreversible fGFR 1–4 inhibitor that shows selective antitumor activity against FGFR-deregulated tumors. Cancer Res.

[45] Meric-Bernstam F, Bahleda R, Hierro C, Sanson M, Bridgewater J, Arkenau HT (2022). Futibatinib, an irreversible FGFR1–4 inhibitor, in patients with advanced solid tumors harboring *FGF/FGFR* aberrations: a phase I dose-expansion study. Cancer Discov.

[46] Meric-Bernstam F, Hollebecque A, Furuse J, Oh D-Y, Bridgewater JA, Shimura M (2023). Management (mgmt) of futibatinib-associated adverse events (AEs) in patients (pts) with advanced cancers: results of a pooled analysis. J Clin Oncol.

[47] Jain RK, Kim Y, Rembisz J, Piekarz R, Synold TW, Zhang J (2022). Phase Ib trial of erdafitinib (E) combined with enfortumab vedotin (EV) following platinum and PD-1/L1 inhibitors for metastatic urothelial carcinoma (mUC) with FGFR2/3 genetic alterations (GAs). J Clin Oncol.

[48] Siefker-Radtke AO, Powles T, Moreno V, Kang TW, Cicin I, Girvin A (2023). Erdafitinib (ERDA) vs ERDA plus cetrelimab (ERDA+CET) for patients (pts) with metastatic urothelial carcinoma (mUC) and fibroblast growth factor receptor alterations (FGFRa): final results from the phase 2 Norse study. J Clin Oncol.

[49] Rosenberg JE, Gajate P, Morales-Barrera R, Lee J-L, Necchi A, Penel N (2020). Safety and preliminary efficacy of rogaratinib in combination with atezolizumab in a phase Ib/II study (FORT-2) of first-line treatment in cisplatin-ineligible patients (pts) with locally advanced or metastatic urothelial cancer (UC) and *FGFR* mRNA overexpression. J Clin Oncol.

[50] Koshkin VS, Sonpavde GP, Hwang C, Mellado B, Tomlinson G, Shimura M (2022). Futibatinib plus pembrolizumab in patients (pts) with advanced or metastatic urothelial carcinoma (mUC): preliminary safety results from a phase 2 study. J Clin Oncol.

[51] Wang Z, Muthusamy V, Petrylak DP, Anderson KS (2023). Tackling FGFR3-driven bladder cancer with a promising synergistic FGFR/HDAC targeted therapy. NPJ Precis Oncol.

[52] Olayioye MA, Neve RM, Lane HA, Hynes NE (2000). The ErbB signaling network: receptor heterodimerization in development and cancer. EMBO J.

[53] Thomas J, Sonpavde G (2022). Molecularly targeted therapy towards genetic alterations in advanced bladder cancer. Cancers (Basel).

[54] Patelli G, Zeppellini A, Spina F, Righetti E, Stabile S, Amatu A (2022). The evolving panorama of HER2-targeted treatments in metastatic urothelial cancer: a systematic review and future perspectives. Cancer Treat Rev.

[55] Moasser MM (2007). The oncogene HER2: its signaling and transforming functions and its role in human cancer pathogenesis. Oncogene.

[56] Ross JS, Wang K, Al-Rohil RN, Nazeer T, Sheehan CE, Otto GA (2014). Advanced urothelial carcinoma: next-generation sequencing reveals diverse genomic alterations and targets of therapy. Mod Pathol.

[57] Zhang H, Berezov A, Wang Q, Zhang G, Drebin J, Murali R (2007). ErbB receptors: from oncogenes to targeted cancer therapies. J Clin Invest.

[58] Tang Y, Zhang X, Qi F, Chen M, Li Y, Liu L (2015). Afatinib inhibits proliferation and invasion and promotes apoptosis of the T24 bladder cancer cell line. Exp Ther Med.

[59] Choudhury NJ, Campanile A, Antic T, Yap KL, Fitzpatrick CA, Wade JL (2016). Afatinib activity in platinum-refractory metastatic urothelial carcinoma in patients with ERBB alterations. J Clin Oncol.

[60] Grivas PD, Day KC, Karatsinides A, Paul A, Shakir N, Owainati I (2013). Evaluation of the antitumor activity of dacomitinib in models of human bladder cancer. Mol Med.

[61] Tsai YC, Ho PY, Tzen KY, Tuan TF, Liu WL, Cheng AL (2015). Synergistic blockade of EGFR and HER2 by new-generation EGFR tyrosine kinase inhibitor enhances radiation effect in bladder cancer cells. Mol Cancer Ther.

[62] Dominguez-Escrig JL, Kelly JD, Neal DE, King SM, Davies BR (2004). Evaluation of the therapeutic potential of the epidermal growth factor receptor tyrosine kinase inhibitor gefitinib in preclinical models of bladder cancer. Clin Cancer Res.

[63] Nutt JE, Lazarowicz HP, Mellon JK, Lunec J (2004). Gefitinib (‘Iressa’, ZD1839) inhibits the growth response of bladder tumour cell lines to epidermal growth factor and induces TIMP2. Br J Cancer.

[64] Miller K, Morant R, Stenzl A, Zuna I, Wirth M (2016). A phase II study of the central european society of anticancer-drug research (cesar) group: results of an open-label study of gemcitabine plus cisplatin with or without concomitant or sequential gefitinib in patients with advanced or metastatic transitional cell carcinoma of the urothelium. Urol Int.

[65] Perrotte P, Matsumoto T, Inoue K, Kuniyasu H, Eve BY, Hicklin DJ (1999). Anti-epidermal growth factor receptor antibody C225 inhibits angiogenesis in human transitional cell carcinoma growing orthotopically in nude mice. Clin Cancer Res.

[66] Inoue K, Slaton JW, Perrotte P, Davis DW, Bruns CJ, Hicklin DJ (2000). Paclitaxel enhances the effects of the anti-epidermal growth factor receptor monoclonal antibody ImClone C225 in mice with metastatic human bladder transitional cell carcinoma. Clin Cancer Res.

[67] Wong YN, Litwin S, Vaughn D, Cohen S, Plimack ER, Lee J (2012). Phase II trial of cetuximab with or without paclitaxel in patients with advanced urothelial tract carcinoma. J Clin Oncol.

[68] Hussain M, Daignault S, Agarwal N, Grivas PD, Siefker-Radtke AO, Puzanov I (2014). A randomized phase 2 trial of gemcitabine/cisplatin with or without cetuximab in patients with advanced urothelial carcinoma. Cancer.

[69] James ND, Liu W, Pirrie S, Kaur B, Hendron C, Ford D (2022). TUXEDO: a phase I/II trial of cetuximab with chemoradiotherapy in muscle-invasive bladder cancer. BJU Int.

[70] Pruthi RS, Nielsen M, Heathcote S, Wallen EM, Rathmell WK, Godley P (2010). A phase II trial of neoadjuvant erlotinib in patients with muscle-invasive bladder cancer undergoing radical cystectomy: clinical and pathological results. BJU Int.

[71] Mohammed A, Miller MS, Lubet RA, Suen CS, Sei S, Shoemaker RH (2020). Combination of erlotinib and naproxen employing pulsatile or intermittent dosing profoundly inhibits urinary bladder cancers. Cancer Prev Res (Phila).

[72] Hussain MH, MacVicar GR, Petrylak DP, Dunn RL, Vaishampayan U, Lara PN Jr (2007). Trastuzumab, paclitaxel, carboplatin, and gemcitabine in advanced human epidermal growth factor receptor-2/*neu*–positive urothelial carcinoma: results of a multicenter phase II national cancer institute trial. J Clin Oncol.

[73] Oudard S, Culine S, Vano Y, Goldwasser F, Théodore C, Nguyen T (2015). Multicentre randomised phase II trial of gemcitabine+platinum, with or without trastuzumab, in advanced or metastatic urothelial carcinoma overexpressing Her2. Eur J Cancer.

[74] Jiang Q, Xie MX, Zhang XC (2020). Complete response to trastuzumab and chemotherapy in recurrent urothelial bladder carcinoma with *HER2* gene amplification: a case report. World J Clin Cases.

[75] Wezel F, Erben P, Gaiser T, Budjan J, von Hardenberg J, Michel MS (2018). Complete and durable remission of human epidermal growth factor receptor 2-positive metastatic urothelial carcinoma following third-line treatment with trastuzumab and gemcitabine. Urol Int.

[76] Hayashi T, Seiler R, Oo HZ, Jäger W, Moskalev I, Awrey S (2015). Targeting HER2 with T-DM1, an antibody cytotoxic drug conjugate, is effective in HER2 over expressing bladder cancer. J Urol.

[77] de Vries EGE, Rüschoff J, Lolkema M, Tabernero J, Gianni L, Voest E (2023). Phase II study (KAMELEON) of single-agent T-DM1 in patients with HER2-positive advanced urothelial bladder cancer or pancreatic cancer/cholangiocarcinoma. Cancer Med.

[78] Meric-Bernstam F, Makker V, Oaknin A, Oh D-Y, Banerjee SN, Gonzalez Martin A (2023). Efficacy and safety of trastuzumab deruxtecan (T-DXd) in patients (pts) with HER2-expressing solid tumors: DESTINY-PanTumor02 (DP-02) interim results. J Clin Oncol.

[79] Powles T, Huddart RA, Elliott T, Sarker SJ, Ackerman C, Jones R (2017). Phase III, double-blind, randomized trial that compared maintenance lapatinib versus placebo after first-line chemotherapy in patients with human epidermal growth factor receptor 1/2–positive metastatic bladder cancer. J Clin Oncol.

[80] Maeda S, Sakai K, Kaji K, Iio A, Nakazawa M, Motegi T (2022). Lapatinib as first-line treatment for muscle-invasive urothelial carcinoma in dogs. Sci Rep.

[81] Gong J, Shen L, Wang W, Fang J (2018). Safety, pharmacokinetics and efficacy of RC48-ADC in a phase I study in patients with HER2-overexpression advanced solid cancer. J Clin Oncol.

[82] Sheng X, Yan X, Wang L, Shi Y, Yao X, Luo H (2021). Open-label, multicenter, phase ii study of RC48-ADC, a HER2-targeting antibody–drug conjugate, in patients with locally advanced or metastatic urothelial carcinoma. Clin Cancer Res.

[83] Zhou L, Xu H, Li S, Yan X, Li J, Wu X (2022). Study RC48-C014: preliminary results of RC48-ADC combined with toripalimab in patients with locally advanced or metastatic urothelial carcinoma. J Clin Oncol.

[84] Sheng X, Zhou L, Yang K, Zhang S, Xu H, Yan X (2023). Disitamab vedotin, a novel humanized anti-HER2 antibody-drug conjugate (ADC), combined with toripalimab in patients with locally advanced or metastatic urothelial carcinoma: an open-label phase 1b/2 study. J Clin Oncol.

[85] Xu Z, Ma J, Chen T, Yang Y (2022). Case report: the remarkable response of pembrolizumab combined with RC48 in the third-line treatment of metastatic urothelial carcinoma. Front Immunol.

[86] Galsky MD, Del Conte G, Foti S, Yu EY, Machiels J-PH, Doger B (2022). Primary analysis from DS8201-A-U105: a phase 1b, two-part, open-label study of trastuzumab deruxtecan (T-DXd) with nivolumab (nivo) in patients (pts) with HER2-expressing urothelial carcinoma (UC). J Clin Oncol.

[87] Peng M, Huang Y, Tao T, Peng CY, Su Q, Xu W (2016). Metformin and gefitinib cooperate to inhibit bladder cancer growth via both AMPK and EGFR pathways joining at Akt and Erk. Sci Rep.

[88] Deng J, Peng M, Wang Z, Zhou S, Xiao D, Deng J (2019). Novel application of metformin combined with targeted drugs on anticancer treatment. Cancer Sci.

[89] Huang Y, Zhou S, He C, Deng J, Tao T, Su Q (2018). Phenformin alone or combined with gefitinib inhibits bladder cancer via AMPK and EGFR pathways. Cancer Commun (Lond).

[90] Ghafouri S, Burkenroad A, Pantuck M, Almomani B, Stefanoudakis D, Shen J (2021). VEGF inhibition in urothelial cancer: the past, present and future. World J Urol.

[91] Narayanan S, Srinivas S (2017). Incorporating VEGF-targeted therapy in advanced urothelial cancer. Ther Adv Med Oncol.

[92] Fus Ł P, Górnicka B (2016). Role of angiogenesis in urothelial bladder carcinoma. Cent European J Urol.

[93] Charlesworth PJ, Harris AL (2006). Mechanisms of disease: angiogenesis in urologic malignancies. Nat Clin Pract Urol.

[94] Nakanishi R, Oka N, Nakatsuji H, Koizumi T, Sakaki M, Takahashi M (2009). Effect of vascular endothelial growth factor and its receptor inhibitor on proliferation and invasion in bladder cancer. Urol Int.

[95] Videira PA, Piteira AR, Cabral MG, Martins C, Correia M, Severino P (2011). Effects of bevacizumab on autocrine VEGF stimulation in bladder cancer cell lines. Urol Int.

[96] Hahn NM, Stadler WM, Zon RT, Waterhouse D, Picus J, Nattam S (2011). Phase II trial of cisplatin, gemcitabine, and bevacizumab as first-line therapy for metastatic urothelial carcinoma: Hoosier Oncology Group GU 04-75. J Clin Oncol.

[97] Rosenberg JE, Ballman KA, Halabi S, Atherton PJ, Mortazavi A, Sweeney C (2021). Randomized phase III trial of gemcitabine and cisplatin with bevacizumab or placebo in patients with advanced urothelial carcinoma: results of CALGB 90601 (Alliance). J Clin Oncol.

[98] Vennepureddy A, Singh P, Rastogi R, Atallah JP, Terjanian T (2017). Evolution of ramucirumab in the treatment of cancer – a review of literature. J Oncol Pharm Pract.

[99] Petrylak DP, Tagawa ST, Kohli M, Eisen A, Canil C, Sridhar SS (2016). Docetaxel as monotherapy or combined with ramucirumab or icrucumab in second-line treatment for locally advanced or metastatic urothelial carcinoma: an open-label, three-arm, randomized controlled phase II trial. J Clin Oncol.

[100] Petrylak DP, de Wit R, Chi KN, Drakaki A, Sternberg CN, Nishiyama H (2020). Ramucirumab plus docetaxel versus placebo plus docetaxel in patients with locally advanced or metastatic urothelial carcinoma after platinum-based therapy (RANGE): overall survival and updated results of a randomised, double-blind, phase 3 trial. Lancet Oncol.

[101] Flaig TW, Su LJ, McCoach C, Li Y, Raben D, Varella-Garcia M (2009). Dual epidermal growth factor receptor and vascular endothelial growth factor receptor inhibition with vandetanib sensitizes bladder cancer cells to cisplatin in a dose- and sequence-dependent manner. BJU Int.

[102] Choueiri TK, Ross RW, Jacobus S, Vaishampayan U, Yu EY, Quinn DI (2012). Double-blind, randomized trial of docetaxel plus vandetanib versus docetaxel plus placebo in platinum-pretreated metastatic urothelial cancer. J Clin Oncol.

[103] Jones R, Crabb S, Chester J, Elliott T, Huddart R, Birtle A (2020). A randomised phase II trial of carboplatin and gemcitabine ± vandetanib in first-line treatment of patients with advanced urothelial cell cancer not suitable to receive cisplatin. BJU Int.

[104] Kumar R, Knick VB, Rudolph SK, Johnson JH, Crosby RM, Crouthamel MC (2007). Pharmacokinetic-pharmacodynamic correlation from mouse to human with pazopanib, a multikinase angiogenesis inhibitor with potent antitumor and antiangiogenic activity. Mol Cancer Ther.

[105] Necchi A, Mariani L, Zaffaroni N, Schwartz LH, Giannatempo P, Crippa F (2012). Pazopanib in advanced and platinum-resistant urothelial cancer: an open-label, single group, phase 2 trial. Lancet Oncol.

[106] Pili R, Qin R, Flynn PJ, Picus J, Millward M, Ho WM (2013). A phase II safety and efficacy study of the vascular endothelial growth factor receptor tyrosine kinase inhibitor pazopanib in patients with metastatic urothelial cancer. Clin Genitourin Cancer.

[107] Gerullis H, Eimer C, Ecke TH, Georgas E, Arndt C, Otto T (2013). Combined treatment with pazopanib and vinflunine in patients with advanced urothelial carcinoma refractory after first-line therapy. Anticancer Drugs.

[108] Narayanan S, Lam A, Vaishampayan U, Harshman L, Fan A, Pachynski R (2016). Phase II study of pazopanib and paclitaxel in patients with refractory urothelial cancer. Clin Genitourin Cancer.

[109] Herbst RS, Arkenau HT, Santana-Davila R, Calvo E, Paz-Ares L, Cassier PA (2019). Ramucirumab plus pembrolizumab in patients with previously treated advanced non-small-cell lung cancer, gastro-oesophageal cancer, or urothelial carcinomas (JVDF): a multicohort, non-randomised, open-label, phase 1a/b trial. Lancet Oncol.

[110] Taylor MH, Lee CH, Makker V, Rasco D, Dutcus CE, Wu J (2020). Phase IB/II trial of lenvatinib plus pembrolizumab in patients with advanced renal cell carcinoma, endometrial cancer, and other selected advanced solid tumors. J Clin Oncol.

[111] Matsubara N, de Wit R, Balar AV, Siefker-Radtke AO, Zolnierek J, Csoszi T (2024). Pembrolizumab with or without lenvatinib as first-line therapy for patients with advanced urothelial carcinoma (LEAP-011): a phase 3, randomized, double-blind trial. Eur Urol.

[112] Bellmunt J, Lalani AA, Jacobus S, Wankowicz SA, Polacek L, Takeda DY (2018). Everolimus and pazopanib (E/P) benefit genomically selected patients with metastatic urothelial carcinoma. Br J Cancer.

[113] Porta C, Giglione P, Liguigli W, Paglino C (2015). Dovitinib (CHIR258, TKI258): structure, development and preclinical and clinical activity. Future Oncol.

[114] Hahn NM, Bivalacqua TJ, Ross AE, Netto GJ, Baras A, Park JC (2017). A phase II trial of dovitinib in BCG-unresponsive urothelial carcinoma with *FGFR3* mutations or overexpression: hoosier cancer research network trial HCRN 12-157. Clin Cancer Res.

[115] Papadopoulos KP, El-Rayes BF, Tolcher AW, Patnaik A, Rasco DW, Harvey RD (2017). A Phase 1 study of ARQ 087, an oral pan-FGFR inhibitor in patients with advanced solid tumours. Br J Cancer.

[116] Meng Z, Pei X, Gu Y, Li L, He D, Wu K (2022). *In vitro* and *in vivo* investigations of anlotinib in bladder cancer treatment. J Clin Oncol.

[117] Qu YY, Sun Z, Han W, Zou Q, Xing N, Luo H (2022). Camrelizumab plus famitinib for advanced or metastatic urothelial carcinoma after platinum-based therapy: data from a multicohort phase 2 study. J Immunother Cancer.

[118] Apolo AB, Nadal R, Tomita Y, Davarpanah NN, Cordes LM, Steinberg SM (2020). Cabozantinib in patients with platinum-refractory metastatic urothelial carcinoma: an open-label, single-centre, phase 2 trial. Lancet Oncol.

[119] Kikuchi E, Hayakawa N (2020). Cabozantinib as a choice for platinum-refractory metastatic urothelial cancer. Lancet Oncol.

[120] Jones RJ, Hussain SA, Birtle AJ, Song YP, Enting D, Faust G (2022). A randomised, double blind, phase II clinical trial of maintenance cabozantinib following chemotherapy for metastatic urothelial carcinoma (mUC): final analysis of the ATLANTIS cabozantinib comparison. J Clin Oncol.

[121] Chaudhry A, Sternberg CN, De Santis M, Bellmunt J, Necchi A, Powles T (2020). FIDES-02, a phase Ib/II study of derazantinib (DZB) as monotherapy and combination therapy with atezolizumab (A) in patients with surgically unresectable or metastaticurothelial cancer (UC) and FGFR genetic aberrations. J Clin Oncol.

[122] Galffy G, Lugowska I, Poddubskaya EV, Cho BC, Ahn MJ, Han JY (2023). A phase II open-label trial of avelumab plus axitinib in previously treated non-small-cell lung cancer or treatment-naïve, cisplatin-ineligible urothelial cancer. ESMO Open.

[123] Apolo AB, Nadal R, Girardi DM, Niglio SA, Ley L, Cordes LM (2020). Phase I Study of Cabozantinib and Nivolumab Alone or With Ipilimumab for Advanced or Metastatic Urothelial Carcinoma and Other Genitourinary Tumors. J Clin Oncol.

[124] Giannatempo P, Guadalupi V, Marandino L, Raggi D, Stellato M, Rametta A (2023). Activity of cabozantinib (CABO) plus durvalumab (DURVA) in patients (pts) with advanced urothelial carcinoma (UC) or non-UC variant histologies (VH) after platinum chemotherapy: interim results from the phase 2 ARCADIA trial. J Clin Oncol.

[125] Pal SK, Agarwal N, Singh P, Necchi A, McGregor BA, Hauke RJ (2022). Cabozantinib (C) in combination with atezolizumab (A) in urothelial carcinoma (UC): results from cohorts 3, 4, 5 of the COSMIC-021 study. J Clin Oncol.

[126] Kilari D, Szabo A, Tripathi A, Paul AK, Alter RS, Bylow KA (2022). A phase 2 study of cabozantinib in combination with atezolizumab as neoadjuvant treatment for muscle-invasive bladder cancer (HCRN GU18-343) ABATE study. J Clin Oncol.

[127] Castellano DE, Duran I, Mellado B, Climent Duran MAA, Garcia del Muro X, Sala González N (2022). Phase I-II study to evaluate safety and efficacy of niraparib plus cabozantinib in patients with advanced urothelial/kidney cancer (NICARAGUA trial): preliminary data of phase I study. J Clin Oncol.

[128] Gupta S, Ballman KV, Galsky MD, Morris MJ, Chen RC, Chan TA (2022). MAIN-CAV: phase III randomized trial of maintenance cabozantinib and avelumab versus avelumab after first-line platinum-based chemotherapy in patients with metastatic urothelial cancer (mUC) (Alliance A032001). J Clin Oncol.

[129] Huan J, Grivas P, Birch J, Hansel DE (2022). Emerging roles for mammalian target of rapamycin (mTOR) complexes in bladder cancer progression and therapy. Cancers (Basel).

[130] Mayer IA, Arteaga CL (2016). The PI3K/akt pathway as a target for cancer treatment. Annu Rev Med.

[131] Bellmunt J, Werner L, Leow JJ, Mullane SA, Fay AP, Riester M (2015). somatic copy number abnormalities and mutations in PI3K/AKT/mTOR pathway have prognostic significance for overall survival in platinum treated locally advanced or metastatic urothelial tumors. PLoS One.

[132] Ma L, Chen Z, Erdjument-Bromage H, Tempst P, Pandolfi PP (2005). Phosphorylation and functional inactivation of TSC2 by Erk implications for tuberous sclerosis and cancer pathogenesis. Cell.

[133] Mendoza MC, Er EE, Blenis J (2011). The Ras-ERK and PI3K-mTOR pathways: cross-talk and compensation. Trends Biochem Sci.

[134] She QB, Halilovic E, Ye Q, Zhen W, Shirasawa S, Sasazuki T (2010). 4E-BP1 is a key effector of the oncogenic activation of the AKT and ERK signaling pathways that integrates their function in tumors. Cancer Cell.

[135] Nazarian R, Shi H, Wang Q, Kong X, Koya RC, Lee H (2010). Melanomas acquire resistance to B-RAF(V600E) inhibition by RTK or N-RAS upregulation. Nature.

[136] Bendell JC, Rodon J, Burris HA, de Jonge M, Verweij J, Birle D (2012). Phase I, dose-escalation study of BKM120, an oral pan-class I PI3K inhibitor, in patients with advanced solid tumors. J Clin Oncol.

[137] McPherson V, Reardon B, Bhayankara A, Scott SN, Boyd ME, Garcia-Grossman IR (2020). A phase 2 trial of buparlisib in patients with platinum-resistant metastatic urothelial carcinoma. Cancer.

[138] Zhu S, Ma AH, Zhu Z, Adib E, Rao T, Li N (2021). Synergistic antitumor activity of pan-PI3K inhibition and immune checkpoint blockade in bladder cancer. J Immunother Cancer.

[139] Farrukh H, Zhu Z, Zhu S, Montgomery RB, Meeks JJ, VanderWeele DJ (2023). A phase II trial with copanlisib plus avelumab as maintenance therapy for metastatic bladder cancer after platinum-based chemotherapy. J Clin Oncol.

[140] Chen CH, Changou CA, Hsieh TH, Lee YC, Chu CY, Hsu KC (2018). Dual inhibition of PIK3C3 and FGFR as a new therapeutic approach to treat bladder cancer. Clin Cancer Res.

[141] Chen CH, Liu YM, Pan SL, Liu YR, Liou JP, Yen Y (2016). Trichlorobenzene-substituted azaaryl compounds as novel FGFR inhibitors exhibiting potent antitumor activity in bladder cancer cells *in vitro* and *in vivo*. Oncotarget.

[142] Juric D, Rodon J, Tabernero J, Janku F, Burris HA, Schellens JHM (2018). Phosphatidylinositol 3-kinase α–selective inhibition with alpelisib (BYL719) in PIK3CA-altered solid tumors: results from the first-in-human study. J Clin Oncol.

[143] Marqués M, Corral S, Sánchez-Díaz M, Del Pozo N, Martínez de Villarreal J, Schweifer N (2023). tumor and stromal cell targeting with nintedanib and alpelisib overcomes intrinsic bladder cancer resistance. Mol Cancer Ther.

[144] Borcoman E, De La Rochere P, Richer W, Vacher S, Chemlali W, Krucker C (2019). Inhibition of PI3K pathway increases immune infiltrate in muscle-invasive bladder cancer. Oncoimmunology.

[145] Zeng SX, Zhu Y, Ma AH, Yu W, Zhang H, Lin TY (2017). The phosphatidylinositol 3-kinase pathway as a potential therapeutic target in bladder cancer. Clin Cancer Res.

[146] De Henau O, Rausch M, Winkler D, Campesato LF, Liu C, Cymerman DH (2016). Overcoming resistance to checkpoint blockade therapy by targeting PI3Kγ in myeloid cells. Nature.

[147] Hong DS, Postow M, Chmielowski B, Sullivan R, Patnaik A, Cohen EEW (2023). Eganelisib, a first-in-class PI3Kγ Inhibitor, in patients with advanced solid tumors: results of the phase 1/1b MARIO-1 trial. Clin Cancer Res.

[148] Sathe A, Guerth F, Cronauer MV, Heck MM, Thalgott M, Gschwend JE (2014). Mutant *PIK3CA* controls DUSP1-dependent ERK 1/2 activity to confer response to AKT target therapy. Br J Cancer.

[149] Dickstein RJ, Nitti G, Dinney CP, Davies BR, Kamat AM, McConkey DJ (2012). Autophagy limits the cytotoxic effects of the AKT inhibitor AZ7328 in human bladder cancer cells. Cancer Biol Ther.

[150] Peng M, Deng J, Zhou S, Xiao D, Long J, Zhang N (2019). Dual inhibition of pirarubicin-induced *AKT* and *ERK* activations by phenformin sensitively suppresses bladder cancer growth. Front Pharmacol.

[151] Kim H, Lee SJ, Lee IK, Min SC, Sung HH, Jeong BC (2020). Synergistic effects of combination therapy with AKT and mTOR inhibitors on bladder cancer cells. Int J Mol Sci.

[152] Isakoff SJ, Tabernero J, Molife LR, Soria JC, Cervantes A, Vogelzang NJ (2020). Antitumor activity of ipatasertib combined with chemotherapy: results from a phase Ib study in solid tumors. Ann Oncol.

[153] Panwalkar A, Verstovsek S, Giles FJ (2004). Mammalian target of rapamycin inhibition as therapy for hematologic malignancies. Cancer.

[154] Mansure JJ, Nassim R, Chevalier S, Rocha J, Scarlata E, Kassouf W (2009). Inhibition of mammalian target of rapamycin as a therapeutic strategy in the management of bladder cancer. Cancer Biol Ther.

[155] Milowsky MI, Iyer G, Regazzi AM, Al-Ahmadie H, Gerst SR, Ostrovnaya I (2013). Phase II study of everolimus in metastatic urothelial cancer. BJU Int.

[156] Iyer G, Hanrahan AJ, Milowsky MI, Al-Ahmadie H, Scott SN, Janakiraman M (2012). Genome sequencing identifies a basis for everolimus sensitivity. Science.

[157] Seront E, Rottey S, Sautois B, Kerger J, D’Hondt LA, Verschaeve V (2012). Phase II study of everolimus in patients with locally advanced or metastatic transitional cell carcinoma of the urothelial tract: clinical activity, molecular response, and biomarkers. Ann Oncol.

[158] Faes S, Demartines N, Dormond O (2017). Resistance to mTORC1 inhibitors in cancer therapy: from kinase mutations to intratumoral heterogeneity of kinase activity. Oxid Med Cell Longev.

[159] Adib E, Klonowska K, Giannikou K, Do KT, Pruitt-Thompson S, Bhushan K (2021). Phase II clinical trial of everolimus in a pan-cancer cohort of patients with mTOR pathway alterations. Clin Cancer Res.

[160] Pan S, Li S, Xiao M, Chen D, Li J (2021). Significant benefit of everolimus in a patient with urothelial bladder cancer harboring a rare M1043I mutation of *PIK3CA*. Invest New Drugs.

[161] Xia W, Zhang S, Duan H, Wang C, Qian S, Shen H (2022). The combination therapy of everolimus and anti-PD-1 improves the antitumor effect by regulating CD8^+^ T cells in bladder cancer. Med Oncol.

[162] Pinto-Leite R, Arantes-Rodrigues R, Ferreira R, Palmeira C, Colaço A, Moreira da Silva V (2014). Temsirolimus improves cytotoxic efficacy of cisplatin and gemcitabine against urinary bladder cancer cell lines. Urol Oncol.

[163] Pulido M, Roubaud G, Cazeau AL, Mahammedi H, Vedrine L, Joly F (2018). Safety and efficacy of temsirolimus as second line treatment for patients with recurrent bladder cancer. BMC Cancer.

[164] Gerullis H, Eimer C, Ecke TH, Georgas E, Freitas C, Kastenholz S (2012). A phase II trial of temsirolimus in second-line metastatic urothelial cancer. Med Oncol.

[165] Chen Y, Zhou X (2020). Research progress of mTOR inhibitors. Eur J Med Chem.

[166] Becker MN, Wu KJ, Marlow LA, Kreinest PA, Vonroemeling CA, Copland JA (2014). The combination of an mTORc1/TORc2 inhibitor with lapatinib is synergistic in bladder cancer *in vitro*. Urol Oncol.

[167] Hernández-Prat A, Rodriguez-Vida A, Juanpere-Rodero N, Arpi O, Menéndez S, Soria-Jiménez L (2019). Novel oral mTORC1/2 inhibitor TAK-228 has synergistic antitumor effects when combined with paclitaxel or PI3Kα inhibitor TAK-117 in preclinical bladder cancer models. Mol Cancer Res.

[168] Voss MH, Gordon MS, Mita M, Rini B, Makker V, Macarulla T (2020). Phase 1 study of mTORC1/2 inhibitor sapanisertib (TAK-228) in advanced solid tumours, with an expansion phase in renal, endometrial or bladder cancer. Br J Cancer.

[169] Kim JW, Milowsky MI, Hahn NM, Kwiatkowski DJ, Morgans AK, Davis NB (2021). Sapanisertib, a dual mTORC1/2 inhibitor, for *TSC1*- or *TSC2*-mutated metastatic urothelial carcinoma (mUC). J Clin Oncol.

[170] Moon du G, Lee SE, Oh MM, Lee SC, Jeong SJ, Hong SK (2014). NVP-BEZ235, a dual PI3K/mTOR inhibitor synergistically potentiates the antitumor effects of cisplatin in bladder cancer cells. Int J Oncol.

[171] Seront E, Rottey S, Filleul B, Glorieux P, Goeminne JC, Verschaeve V (2016). Phase II study of dual phosphoinositol-3-kinase (PI3K) and mammalian target of rapamycin (mTOR) inhibitor BEZ235 in patients with locally advanced or metastatic transitional cell carcinoma. BJU Int.

[172] Hyman DM, Tran B, Paz-Ares L, Machiels J-P, Schellens JH, Bedard PL (2019). Combined PIK3CA and fgfr inhibition with alpelisib and infigratinib in patients with PIK3CA-mutant solid tumors, with or without FGFR alterations. JCO Precis Oncol.

[173] Braicu C, Buse M, Busuioc C, Drula R, Gulei D, Raduly L (2019). A comprehensive review on MAPK: a promising therapeutic target in cancer. Cancers (Basel).

[174] Necchi A, Madison R, Pal SK, Ross JS, Agarwal N, Sonpavde G (2021). Comprehensive genomic profiling of upper-tract and bladder urothelial carcinoma. Eur Urol Focus.

[175] Rosenberg JE, von der Maase H, Seigne JD, Mardiak J, Vaughn DJ, Moore M (2005). A phase II trial of R115777, an oral farnesyl transferase inhibitor, in patients with advanced urothelial tract transitional cell carcinoma. Cancer.

[176] Lee HW, Sa JK, Gualberto A, Scholz C, Sung HH, Jeong BC (2020). A phase II trial of tipifarnib for patients with previously treated, metastatic urothelial carcinoma harboring HRAS mutations. Clin Cancer Res.

[177] Rose A, Grandoch M, vom Dorp F, Rübben H, Rosenkranz A, Fischer JW (2010). Stimulatory effects of the multi-kinase inhibitor sorafenib on human bladder cancer cells. Br J Pharmacol.

[178] Dreicer R, Li H, Stein M, DiPaola R, Eleff M, Roth BJ (2009). Phase 2 trial of sorafenib in patients with advanced urothelial cancer: a trial of the Eastern Cooperative Oncology Group. Cancer.

[179] Shah CH, Pappot H, Agerbæk M, Holmsten K, Jäderling F, Yachnin J (2019). Safety and Activity of sorafenib in addition to vinflunine in post‐platinum metastatic urothelial carcinoma (vinsor): phase I trial. Oncologist.

[180] Necchi A, Lo Vullo S, Raggi D, Perrone F, Giannatempo P, Calareso G (2018). Neoadjuvant sorafenib, gemcitabine, and cisplatin administration preceding cystectomy in patients with muscle-invasive urothelial bladder carcinoma: an open-label, single-arm, single-center, phase 2 study. Urol Oncol.

[181] Bhattacharjee S, Sullivan MJ, Wynn RR, Demagall A, Hendrix AS, Sindhwani P (2022). PARP inhibitors chemopotentiate and synergize with cisplatin to inhibit bladder cancer cell survival and tumor growth. BMC Cancer.

[182] Grivas P, Loriot Y, Morales-Barrera R, Teo MY, Zakharia Y, Feyerabend S (2021). Efficacy and safety of rucaparib in previously treated, locally advanced or metastatic urothelial carcinoma from a phase 2, open-label trial (ATLAS). BMC Cancer.

[183] Crabb SJ, Hussain S, Soulis E, Hinsley S, Dempsey L, Trevethan A (2023). A randomized, double-blind, biomarker-selected, phase II clinical trial of maintenance poly adp-ribose polymerase inhibition with rucaparib following chemotherapy for metastatic urothelial carcinoma. J Clin Oncol.

[184] Doroshow DB, O’Donnell PH, Hoffman-Censits JH, Gupta SV, Vaishampayan U, Heath EI (2023). Phase II trial of olaparib in patients with metastatic urothelial cancer harboring DNA damage response gene alterations. JCO Precis Oncol.

[185] Rodriguez-Moreno JF, de Velasco G, Alvarez-Fernandez C, Collado R, Fernandez-Rodriguez R, Estevez SV (2020). 761P Impact of the combination of durvalumab (MEDI4736) plus olaparib (AZD2281) administered prior to surgery in the molecular profile of resectable urothelial bladder cancer. NEODURVARIB trial. Ann Oncol.

[186] Rosenberg JE, Park SH, Kozlov V, Dao TV, Castellano D, Li J-R (2022). Durvalumab Plus Olaparib in Previously Untreated, Platinum-Ineligible Patients With Metastatic Urothelial Carcinoma: A Multicenter, Randomized, Phase II Trial (BAYOU). J Clin Oncol.

[187] Vignani F, Tambaro R, De Giorgi U, Giannatempo P, Bimbatti D, Carella C (2023). Addition of niraparib to best supportive care as maintenance treatment in patients with advanced urothelial carcinoma whose disease did not progress after first-line platinum-based chemotherapy: the meet-URO12 randomized phase 2 trial. Eur Urol.

[188] Yap TA, Bardia A, Dvorkin M, Galsky MD, Beck JT, Wise DR (2023). Avelumab plus talazoparib in patients with advanced solid tumors: the JAVELIN PARP medley nonrandomized controlled trial. JAMA Oncol.

[189] Coquan E, Clarisse B, Lequesne J, Brachet PE, Nevière Z, Meriaux E (2022). TALASUR trial: a single arm phase II trial assessing efficacy and safety of TALazoparib and avelumab as maintenance therapy in platinum-sensitive metastatic or locally advanced URothelial carcinoma. BMC Cancer.

[190] Quinn DI, Tsao-Wei DD, Twardowski P, Aparicio AM, Frankel P, Chatta G (2021). Phase II study of the histone deacetylase inhibitor vorinostat (suberoylanilide hydroxamic acid; SAHA) in recurrent or metastatic transitional cell carcinoma of the urothelium – an NCI-CTEP sponsored: california cancer consortium trial, NCI 6879. Invest New Drugs.

[191] Buckley MT, Yoon J, Yee H, Chiriboga L, Liebes L, Ara G (2007). The histone deacetylase inhibitor belinostat (PXD101) suppresses bladder cancer cell growth *in vitro* and *in vivo*. J Transl Med.

[192] Hussain MHA, Kocherginsky M, Singh P, Myint Z, Jiang DM, Wulff-Burchfield EM (2023). A pilot study of tazemetostat and pembrolizumab in advanced urothelial carcinoma (ETCTN 10183). J Clin Oncol.

[193] Garnett MJ, Edelman EJ, Heidorn SJ, Greenman CD, Dastur A, Lau KW (2012). Systematic identification of genomic markers of drug sensitivity in cancer cells. Nature.

[194] Rose TL, Chism DD, Alva AS, Deal AM, Maygarden SJ, Whang YE (2018). Phase II trial of palbociclib in patients with metastatic urothelial cancer after failure of first-line chemotherapy. Br J Cancer.

[195] Klein FG, Granier C, Zhao Y, Pan Q, Tong Z, Gschwend JE (2021). Combination of talazoparib and palbociclib as a potent treatment strategy in bladder cancer. J Pers Med.

[196] Mariathasan S, Turley SJ, Nickles D, Castiglioni A, Yuen K, Wang Y (2018). TGFβ attenuates tumour response to PD-L1 blockade by contributing to exclusion of T cells. Nature.

[197] McGovern K, Castro A, Cavanaugh J, Sanchez-Martin M, Tchaicha J, Syed S (2020). 448 Discovery of clinical candidate IK-175, a selective orally active AHR antagonist. J Immunother Cancer.

[198] Aggen D, McKean M, Lakhani N, Bashir B, Hoffman-Censits J, Alhalabi O (2022). 661 Initial results from a phase 1a/b study of IK-175, an oral AHR inhibitor, as a single agent and in combination with nivolumab in patients with advanced solid tumors and urothelial carcinoma. J Immunother Cancer.

